# Structural and Biological Investigations for a Series of N-5 Substituted Pyrrolo[3,2-*d*]pyrimidines as Potential Anti-Cancer Therapeutics

**DOI:** 10.3390/molecules24142656

**Published:** 2019-07-23

**Authors:** Brian M. Cawrse, Nia’mani M. Robinson, Nina C. Lee, Gerald M. Wilson, Katherine L. Seley-Radtke

**Affiliations:** 1 Department of Chemistry and Biochemistry, University of Maryland, Baltimore County, Baltimore, MD 21250, USA; 2 Department of Biochemistry and Molecular Biology, University of Maryland School of Medicine, Baltimore, MD 21201, USA

**Keywords:** pyrrolo[3,2-*d*]pyrimidine, anti-cancer, antiproliferative, DNA damage, DNA alkylator

## Abstract

Pyrrolo[3,2-*d*]pyrimidines have been studied for many years as potential lead compounds for the development of antiproliferative agents. Much of the focus has been on modifications to the pyrimidine ring, with enzymatic recognition often modulated by C2 and C4 substituents. In contrast, this work focuses on the N5 of the pyrrole ring by means of a series of novel N5-substituted pyrrolo[3,2-*d*]pyrimidines. The compounds were screened against the NCI-60 Human Tumor Cell Line panel, and the results were analyzed using the COMPARE algorithm to elucidate potential mechanisms of action. COMPARE analysis returned strong correlation to known DNA alkylators and groove binders, corroborating the hypothesis that these pyrrolo[3,2-*d*]pyrimidines act as DNA or RNA alkylators. In addition, N5 substitution reduced the EC_50_ against CCRF-CEM leukemia cells by up to 7-fold, indicating that this position is of interest in the development of antiproliferative lead compounds based on the pyrrolo[3,2-*d*]pyrimidine scaffold.

## 1. Introduction

**Figure 1:**
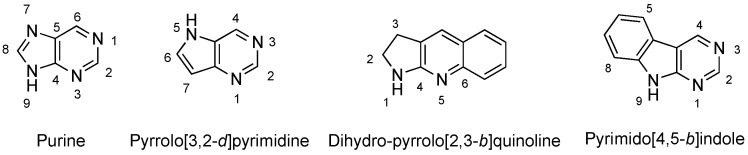
Structures of biologically relevant heterocycles.

Pyrrolo[3,2-*d*]pyrimidines, or 9-deazapurines, represent a class of compounds that are sterically and electronically similar to the naturally occurring purine nucleobases, with the exception of a hydrogen-bond donating moiety at N5 (Figure 1) [1,2,3]. These compounds have seen widespread biological activity and have been extensively utilized in the design of small molecule inhibitors of purine nucleoside phosphorylase (PNP) [4,5,6,7,8,9], dihydrofolate reductase (DHFR) [3], the transient receptor potential channel family [10], and various kinases [11,12,13]. In addition, pyrrolo[3,2-*d*]pyrimidines have shown promise in the development of antitumor agents [1,14,15,16]. The research detailed herein examined the effect of N5 substitution on the pyrrolo[3,2-*d*]pyrimidine scaffold, with ten novel compounds synthesized and subsequently tested for antiproliferative activity.

N5 and C7 substitutions have been shown by multiple researchers to play important roles in the potency of pyrrolo[3,2-*d*]pyrimidines designed as enzyme inhibitors and potential therapeutics. Recently, the pyrrolo[3,2-*d*]pyrimidine scaffold has been shown to inhibit Multi-drug Resistance-Associated Protein 1 (MRP1), an active efflux transporter responsible for resistance to a wide variety of antiproliferative agents [1,17]. Similarly, multiple quinazolinone scaffolds have been investigated as potent inhibitors of MRP1 [18,19], which led to the development of dihydropyrroloquinoline [20], indolopyrimidine [21], and pyrrolopyrimidine [1,17,18,21] inhibitors (Figure 1).

**Figure 2:**
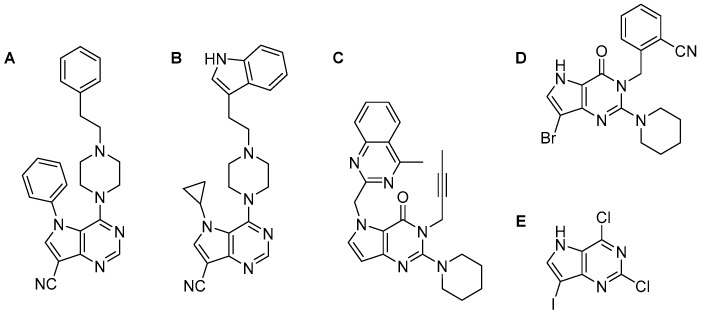
Structures of active pyrrolo[3,2-*d*]pyrimidines. (**A** and **B**) Inhibitors of multidrug resistance protein 1 (MRP1), P-glycoprotein, and breast cancer resistance protein (BCRP). (**C** and **D**) Inhibitors of dipeptidyl peptidase IV (DPP-IV). (**E**) Broad-spectrum antiproliferative agent, with potent activity against MIA PaCa-2 pancreatic cells.

Schmitt et al. synthesized a large series of 7-cyano-pyrrolo[3,2-*d*]pyrimidines that mapped the effects of various substituents at C4, N5, and C6 [1,17,22]. Their findings suggested that an aliphatic group at N5 increased activity against MRP1, but this activity was not further increased by the addition of aromatic rings at the end of the aliphatic N5 chains (Figure 2**A**) [22]. A second study by the same group verified the increase in MRP1 activity with N5 aliphatic substitutions, with a cyclopropyl group imparting the greatest effect (Figure 2**B**) [17]. 

Zeng et al. synthesized C2/N3 substituted pyrrolo[3,2-*d*]pyrimidine inhibitors of dipeptidyl peptidase IV (DPP-IV) that explored the effect of various quinolone and quinazoline substituents, finding that different N5 bicyclic substituents had widely differential activities (1.5–139 nM, Figure 2**C**) [23]. Related to this, Xie et al. synthesized N2/N3 substituted pyrrolo[3,2-*d*]pyrimidines as potent and selective inhibitors of dipeptidyl peptidase IV (DPP-IV) that explored the effect of C7 substitution, finding that C7-bromine imparted the greatest desired activity (Figure 2**D**) [24]. 

Finally, Temburnikar et al. previously determined that 2,4-dichloro-pyrrolo[3,2-*d*]pyrimidines showed broad-spectrum antiproliferative activity, which was greatly enhanced via C7 halogenation (Figure 2**E**) [16,25,26]. In addition, N5 substitution led to a marked decrease in toxicity, lending credence to the hypothesis that the toxicity of these halogenated pyrrolo[3,2-*d*]pyrimidines can be modulated in order to take full advantage of their potent antiproliferative properties [15]. 

**Figure 3:**
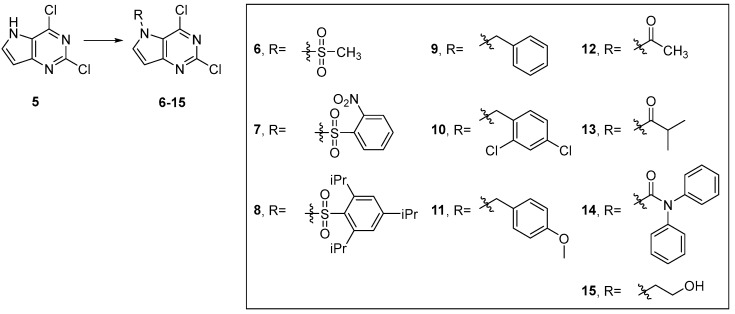
Target compounds, starting from parent compound **5**. Compounds **6**–**15** were submitted to the National Cancer Institute for NCI-60 cell line screening.

These studies all suggested that the N5 position of pyrrolo[3,2-*d*]pyrimidines was of interest in potentially improving activity and modulating toxicity. As a result, a series of N5 substituted compounds were synthesized and characterized as part of an ongoing structure-activity relationship study, designed to investigate the effect of various substituents at N5. The target compounds are shown in Figure 3. The sulfonamide compounds **6**–**8** are a continuation of a previous study that indicated that N5 sulfonamide substituents decreased toxicity without adversely affecting activity [15]. The benzyl compound series **9**–**11** provided an opportunity to explore whether the N5 substituent requires hydrolytic cleavage prior to antiproliferative activity, as was indicated in earlier pharmacokinetic testing [15]. Amide substitution products **12**–**14** represent a series of compounds possessing the naturally occurring and medicinally prevalent amide group. Finally, the addition of a polar aliphatic alcohol decreased log P from 1.66 for parent compound **5** to 1.38 for compound **15**, allowing investigation of the effects of increased aqueous solubility. 

The most active compounds from the NCI-60 cell line screen (**7**, **9**, **10**, and **14**) were subjected to cell assays to determine their activity against the cell line CCRF-CEM, an acute lymphoblastic leukemia cell line, derived from T lymphoblast cells [27]. Finally, chemical and pharmacokinetic properties were calculated using Schrodinger QikProp software (Schrodinger, LLC., New York, NY, USA) [28]. 

## 2. Results

### 2.1. National Cancer Institute-60 Human Tumor Cell Line Screen and COMPARE Analysis

In 1990, the National Cancer Institute (NCI) established a 60 human cell line screen for the evaluation of antiproliferative agents [29,30,31]. Represented cell lines encompass leukemia, melanoma, lung, colon, nervous system, ovarian, renal, prostate, and breast cancers. Compounds **6**–**15** were screened at 10 µM concentrations, and the results indicated that N5 substitution has a significant effect on both activity and selectivity. The results are presented in terms of growth percent (GP), which is the growth of a treated culture compared to the growth of an untreated culture, i.e., 60% GP represents 40% growth inhibition, compared to a negative control cell culture [30]. GP between 0–99 represents cytostatic properties; between −100–0 represents cytotoxic properties [30]. Table 1 shows the best growth percent for each compound, which represents the maximal inhibitory effect against the most affected cell line. The complete NCI-60 data can be found in the Supplemental Information. 

Overall, the compounds were minimally active as broad-spectrum antiproliferatives, but did have specificity leukemia cell lines, with good activity for compounds **7**, **9**, **10**, and **14**. Compound **7** affected the cell growth in leukemia (CCRF-CEM, K-562, and RPMI-8226) cell lines, with an average GP of 63.6% (Supplemental Information). Compound **7** showed best growth inhibition against NCI-H522 non-small cell lung cancer (49.5% GP) but did not affect other non-small cell lung cancer lines. The two other sulfonyl substituted compounds (**6** and **8**) did not show significant activity. Benzyl substituted compounds **9**–**11** showed the best activity against the CCRF-CEM leukemia cell line, with GP ranging from 22.88–65.83 (Table 1). Compound **10** also displayed potent activity against NCI-H522 non-small cell lung cancer, with a 35.1% GP (Supplemental Information). Within the amide substituted compounds **12**–**14**, only **14** showed appreciable activity, and in general, this compound was the most potent. Notably, this compound was the only one to show lethality against breast cancer cell lines, with −0.95% GP against MDA-MB-468 (Table 1). Finally, hydroxyethyl substituted **15** showed decreased activity compared to parent **5**. 

COMPARE analysis [31,32,33,34,35,36] was then performed for compounds **5**–**15** against the NCI Standard Agents list, an aggregate of 171 compounds that have shown clinical antiproliferative activity. The analysis returns a Pearson correlation coefficient (PCC; [37,38]) that ranges from −1.0 to 1.0, representing the statistical similarity of a compound, compared to the activity of the known standard with 1.0 representing a perfect match, and −1.0 representing a perfect mirror image of the compared activities [31]. Moderate correlation is represented by PCC values between 0.5–0.7, and strongly analogous compounds generally have a PCC of ≥ 0.7 [39]. The top five correlation results all had PCC ≥ 0.6, indicating a moderate correlation (Table 2).

### 2.2. In Vitro Cell Growth Inhibition

**Figure 4:**
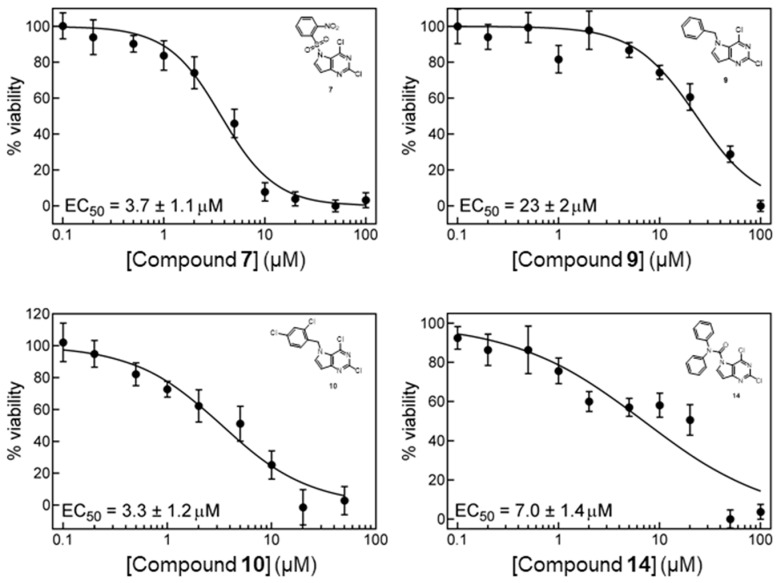
CCRF-CEM MTT assay results.

Based on the results of the NCI-60 screen, compounds **7**, **9**, **10**, and **14** were selected for in vitro assays against CCRF-CEM leukemia cells, using a commercially available MTT assay kit [27]. The most active compound in this assay was **10**, with an EC_50_ = 3.3 ± 1.2 µM; the least active compound was **9**, with EC_50_ = 23.2 ± 2.0 µM. The single digit micromolar EC_50_ values observed for **7**, **10**, and **14** represent an approximately 7-fold increase in activity compared to parent unsubstituted compound **5**, which has low activity against CCRF-CEM cells (EC_50_^CCRF-CEM^ = 25 ± 2 µM) [16]. The results of the contemporary assays are shown in Figure 4.

## 3. Discussion

Previous studies on pyrrolo[3,2-*d*]pyrimidines indicate that the scaffold holds promise in the development of potent antiproliferative agents [16]. Halogenated pyrrolo[3,2-*d*]pyrimidines, synthesized previously by the Seley-Radtke laboratory, exhibited low micromolar to nanomolar activity against a wide variety of cancer cell lines, with results indicating that N5 substitution allows modulation of toxicity and activity [15,16]. The SAR study presented here corroborated those results, with NCI-60 cell line screening showing good activity and specificity against CCRF-CEM leukemia. 

The results observed for compounds **9**–**11** strongly suggest that enzymatic cleavage of the N5 substituent is not required for biological activity, as the benzyl substituents are assumed to be stable under physiological conditions. Interestingly, this series showed the best activity as a group, with preferential inhibition in leukemia cultures. Leukemia cells have been associated with an abnormally high rate of cell proliferation (cell birth rates have been measured at 0.07–1.31% per day), as well as a low rate of cell death [40,41]. In addition, acute myeloid leukemia has been demonstrated to have an increased expression of non-coding RNA (miRNA), associated with leukemia pathogenesis and leukemogenesis, providing additional nucleic acid targets for DNA/RNA alkylating molecules [42,43]. This correlates with the trend for preferential leukemia growth inhibition observed for **9**–**11**.

The necessity for aromatic substituents was further demonstrated in the acyl/amide substituted compounds **12**–**14**, with the acetyl- and isobutyryl-substituted compounds **12** and **13** showing little growth inhibition, compared to the significant inhibitory activity of *N*,*N*-diphenylcarboxamide **14**. Compound **14** showed the greatest inhibitory activity overall, suggesting that the single aromatic ring present in **7**–**11** may not be sufficient to impart maximal inhibitory activity. 

Hydroxyethyl substituted **15** showed the least inhibitory action of all of the compounds tested, suggesting that increased hydrophilicity is not a factor in the activity of these compounds.

COMPARE analysis returned moderate to strong correlation to d-tetrandrine, pibenzimol hydrochloride, fluorodopan, melphalan, and carmustine. d-tetrandrine is a naturally occurring benzylisoquinoline alkaloid that has been shown to associate with DNA and RNA in sarcoma cell lines [44]; pibenzimol HCl is a benzimidazole DNA minor groove binder and inhibitor of topoisomerase I [45,46]; fluorodopan is a halogenated pyrimidine-2,4-dione once considered as a possible treatment for colorectal cancer [47]; melphalan and carmustine are both alkylating nitrogen mustards used to treat multiple myeloma, and glioblastoma, respectively [48,49]. These compounds all share a similar mechanism of action—direct association or alkylation of nucleic acids, suggesting that compounds **6**–**15** may follow a similar mechanism. This observation supports previous studies that indicated the compounds operate via an alkylation mechanism. Briefly, flow cytometry revealed an accumulation in the G2/M phase, consistent with a DNA damage checkpoint, and γ-H2Ax staining of cell cultures treated with an analogue of parent **5** revealed DNA damage [15,16]. In addition, a proteomic study did not reveal any covalent protein targets, which suggested that the inhibition of enzymes such as topoisomerases is unlikely (unpublished results).

The in vitro cell proliferation assays showed that compound **10** was the most efficacious at preventing cell proliferation, with an EC_50_ = 3.3 ± 1.2 µM. Compound **9** was the least effective, with an EC_50_ = 23 ± 2 µM. The low EC_50_ observed for compound **9** may be due to the chemical properties of this compound, in particular the solvent accessible surface area (SASA) and electron affinity (EA). Of the four compounds tested, **9** has the lowest SASA and EA, which would hinder the bioavailability and ability to accept an electron during an alkylation reaction, (Table 3) [50]. However, compared to the parent compound **5** (2,4-dichloro-5H-pyrrolo[3,2-*d*]pyrimidine), compounds **7**, **10**, and **14** all showed better activity in CCRF-CEM cells by up to a factor of ~7 (EC_50_^CCRF-CEM^ = 25 ± 2 µM for compound **5**) [16], indicating that N5 substitution may offer a viable path for development of potent antiproliferative lead compounds.

In summary, the structure-activity relationship study of compounds **6**–**15** revealed that aromatic substitutions impart increased activity, with a trend towards inhibition of leukemia. The addition of a small polar substituent (compound **15**) resulted in activity similar to that of the parent compound **5**, suggesting that hydrophilicity does not affect activity. These compounds do not appear to have a particular protein target, and instead mirror the activity of known DNA alkylators and groove binders. This is in line with earlier findings of DNA damage by analogues of parent compound **5**. In vitro assays show low micromolar EC_50_ values for three of the four most active compounds, which represents a 7-fold increase in activity, compared to the unsubstituted parent compound**.** Together, these data support that halogenated pyrrolo[3,2-*d*]pyrimidines act as non-specific antiproliferative agents with tunable activity via N5 substitution, and that further study of N5 substituents holds the potential to improve activity and cell line specificity.

## 4. Materials and Methods

### 4.1. NCI-60 Human Tumor Cell Lines Screen and COMPARE Analysis

The standard operating procedures for the NCI-60 cell line screen have been well-documented and reported in multiple sources [29,30,33,36]. Briefly, accepted antiproliferative compounds are dissolved in DMSO, then stored frozen prior to use. All cultures are grown in RPMI 1640 medium, supplemented with 5% bovine serum albumin and 2 mM l-glutamine. Prior to evaluation, cultures are dispensed into 96-well microtiter plates at densities between 5000–40,000 cells/well, then incubated for 24 h at 37 °C, 5% CO_2_, 95% air, 100% humidity. Time zero cell density is determined by fixing two wells with trichloroacetic acid (TCA). Experimental compounds are diluted to 10 µM in complete medium containing 50 µg/mL gentamicin and administered to plate wells. Plates are incubated for 48 h, followed by addition of TCA to a final concentration of 10% and held at 60 °C for 4 h. Supernatant is discarded, and protein content of wells is determined by sulforhodamine B staining. Percentage growth inhibition is determined relative to DMSO-treated cells and time-zero control wells. A list of current cell lines included in the NCI-60 screen can be found at https://dtp.cancer.gov/discovery_development/nci-60/cell_list.htm, and is also detailed in the NCI-60 data in the Supplementary Information.

COMPARE analysis was performed using the publicly available online COMPARE algorithm [51]. Using compounds **6**–**15** as seed compounds, and by comparing to the NCI Standard Agents list, growth inhibition patterns can be compared across the NCI-60 screening results. The results were quantitated as a Pearson correlation coefficient (PCC) with high-ranking compounds, suggesting a mechanism of action similar to that of the seed compounds [33]. Parameters selected for analysis were: Target Set Name: Standard Agents; Target Set Endpoint: GI_50_; Minimum Correlation: 0.4; Minimum Count Common Cell Lines: 40; Minimum Standard Deviation: 0.05.

### 4.2. In Vitro Cell Growth Inhibition

A total of 4 mM stock solutions of compounds **7**, **9**, **10**, and **14** were prepared in DMSO to be used in serial dilutions to achieve final concentrations of 0, 0.1, 0.2, 0.5, 1, 2, 5, 10, 20, 50, and 100 µM in cell-seeded 96-well plates. Human lymphocytic CCRF-CEM cells were obtained from the American Type Culture Collection (ATCC, Rockville, MD, USA) and cultured in RPMI 1640 (Corning Life Sciences, Tewksbury, MA, USA) containing 1× penicillin/streptomycin (Corning) and 10% fetal calf serum (Sigma-Aldrich, St. Louis, MO, USA) at 37 °C in a humidified, CO_2_-controlled incubator. The cytostatic assays were performed by incubating 5 × 10^4^ CCRF-CEM cells in 0.1 mL media with the described concentration of test compounds or vehicle (DMSO) for 48 h. Cell viability was then measured using the MTT Cell Proliferation Assay kit (ATCC) according to the manufacturer’s instructions, with absorbance measured at 595 nm. Percent cell viability was calculated relative to cells incubated with vehicle alone, then plotted as a function of drug concentration. EC_50_ values were resolved by analyzing viability curves using a 4-parameter dose response function utilizing PRISM v3.03 software (GraphPad, San Diego, CA, USA).

### 4.3. Chemistry

#### 4.3.1. General Experimental

All solvents and reagents purchased through Fisher Scientific (Hampton, NH, USA) or Sigma-Aldrich (St. Louis, MO, USA). All ^1^H- and ^13^C-NMR were obtained on a JEOL ECX 400 MHz NMR. All NMR spectra were referenced to tetramethylsilane (TMS) at 0.0 ppm. The spin multiplicities are indicated by the symbols s (singlet), d (doublet), dd (doublet of doublets), t (triplet), q (quartet), m (multiplet), and br (broad). All NMR solvents were obtained from Cambridge Isotope Laboratories. All reactions were monitored by thin-layer chromatography (TLC) on 0.25 mm precoated silica glass plates. All column chromatography was run 32–63 micron silica gel obtain from Dynamic Adsorptions Inc. (Norcross, GA, USA). Yields refer to chromatographically and spectroscopically homogenous materials. All mass spectra were recorded and obtained from the UMBC Mass Spectrometry Facility using a Bruker amazon Speed Ion Trap fitted with an electrospray (ESI) or atmospheric pressure chemical ionization (APCI) injection port. Elemental analysis was conducted by Atlantic Microlabs (Norcross, GA, USA) via combustion analysis.

#### 4.3.2. Synthesis of Target Compound **5**

**Scheme 1:**
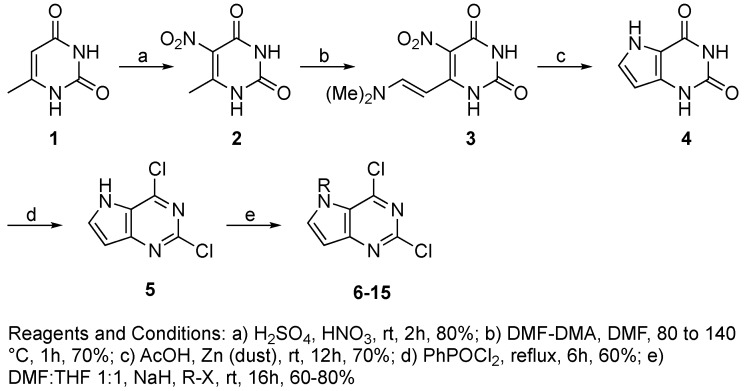
Synthesis of parent 2,4-dichloro-pyrrolo[3,2-*d*]pyrimidine **5** and N5 substituted analogues **6**–**15**.

Parent compound **5** was synthesized starting from commercially available 6-methyluracil (**1**), shown in Scheme 1. Nitration at C5 was accomplished by adding 6-methyluracil to a solution of concentrated sulfuric and nitric acids at 0 °C, followed by quenching in ice water to give **2** as a pale yellow solid [24]. This was followed by a modified Batcho-Leimgruber indole synthesis to first prepare enamine **3** [52,53]. Ring cyclization is accomplished by reduction of the nitro moiety to an amino group, with subsequent nucleophilic attack at C8 by the amino lone pair with loss of dimethylamine. The reduction of the nitro group is accomplished by stirring **3** in acetic acid and zinc dust overnight at 80 °C, followed by filtration, formation of the N5 sodium salt via NaOH dissolution, then re-acidification and collection of solid **4** [23,54].

The parent compound **5** was synthesized by first reforming the sodium salt of **4**, by stirring compound **4** in 1 M NaOH at 40 °C for one hour, then followed by crystallization at 0 °C and repeated washes with cold water and acetone. Chlorination of the sodium salt of **4** was achieved by refluxing for 5 h in phenylphosphonic dichloride under a nitrogen atmosphere. Substitution at N5 was accomplished by N5 deprotonation using sodium hydride at 0 °C, followed by addition of the appropriate halogenated reagent to obtain target compounds **6**–**15**.

#### 4.3.3. General Procedure for Synthesis of Compounds **6**–**15**

2,4-dichloro-1H-pyrrolo[3,2-*d*]pyridine (**5**) was dissolved in a solution of 1:1 THF:DMF under N_2_. Sodium hydride (60% suspension in oil) was added directly, and the mixture was stirred at room temperature for 1 h. Appropriate halogenated reagent (methanesulfonyl chloride (**6**), 2-nitrobenzenesulfonyl chloride (**7**), 2,4,6-Triisopropylbenzenesulfonyl chloride (**8**), benzyl bromide (**9**), 2,4-dichlorobenzyl bromide (**10**), 4-methoxybenzyl chloride (**11**), acetyl chloride (**12**), isobutyryl chloride (**13**), diphenylcarbamyl chloride (**14**), iodoethane (**15**)) was added and the mixture was stirred at r.t. for 18 h. The solvent was removed and residue dissolved in EtOAc and washed with water and brine, then dried over MgSO_4_. Organics were loaded onto Celite and product purified using silica column chromatography eluting with 4:1 hexanes:EtOAc or 9:1 DCM:MeOH to obtain product, generally as a white to off-white solid.

#### 4.3.4. Characterization

*2,4-dichloro-5-(methylsulfonyl)-5H-pyrrolo[3,2-d]pyrimidine* (**6**). ^1^H-NMR (400 MHz, CDCl_3_) (ppm) δ: 3.66 (s, 3H); 6.83 (d, *J* = 4.12 Hz, 1H); 8.10 (d, *J* = 4.12 Hz, 1H). ^13^C-NMR (100 MHz, DMSO-*d*_6_): δ158.79, 153.40, 152.14, 144.44, 139.10, 137.58, 124.23, 123.05, 105.97, 102.31, 44.88. ESI-MS *m*/*z* for C_7_H_5_Cl_2_N_3_O_2_S calcd. at 264.95, found at 266.01 [M + H]^+^. Anal. calcd. for C_7_H_5_Cl_2_N_3_O_2_S: C, 31.60; H, 1.89; N, 15.79. Found: C, 31.58; H, 1.89; N, 15.78.

*2,4-dichloro-5-((2-nitrophenyl)sulfonyl)-5H-pyrrolo[3,2-d]pyrimidine* (**7**). ^1^H-NMR (400 MHz, DMSO-*d*_6_) (ppm) δ: 7.20 (t, *J* = 2.32 Hz, 1H); 7.75 (d, *J* = 8.24 Hz, 1H); 7.82 (t, *J* = 7.80 Hz, 1H); 8.04 (q, *J* = 7.32 Hz, 1H); 8.25 (d, *J* = 7.80 Hz, 1H); 8.60 (d, *J* = 2.76 Hz, 1H). ^13^C-NMR (100 MHz, DMSO-*d*_6_): δ158.30, 152.97, 147.51, 144.41, 140.13, 137.28, 134.88, 131.37, 130.61, 126.68, 108.04, 102.79. ESI-MS *m*/*z* for C_12_H_6_Cl_2_N_4_O_4_S calcd. at 371.95, found at 373.00 [M + H]^+^. Anal. calcd. for C_12_H_6_Cl_2_N_4_O_4_S: C, 38.62; H, 1.62; N, 15.01. Found: C, 38.61; H, 1.62; N, 15.01.

*2,4-dichloro-5-((2,4,6-triisopropylphenyl)sulfonyl)-5H-pyrrolo[3,2-d]pyrimidine* (**8**). ^1^H-NMR (400 MHz, CDCl_3_) (ppm) δ: 1.08 (d, *J* = 6.84 Hz, 12H); 1.24 (d, *J* = 6.88 Hz, 6H); 2.92 (septet, 1H); 3.85 (septet, 2H); 6.83 (d, *J* = 3.64 Hz, 1H); 7.17 (s, 2H); 8.26 (d, *J* = 3.68 Hz, 1H). ^13^C-NMR (100 MHz, CDCl_3_): δ157.78, 155.99, 153.18, 151.22, 145.17, 136.98, 131.89, 124.12, 123.62, 105.89, 34.42, 30.26, 24.38, 23.55. ESI-MS *m*/*z* for C_21_H_25_Cl_2_N_3_O_2_S calcd. at 453.10, found at 454.10 [M + H]^+^. Anal. calcd. for C_21_H_25_Cl_2_N_3_O_2_S: C, 55.51; H, 5.55; N, 9.25. Found: C, 55.49; H, 5.54; N, 9.24.

*2,4-dichloro-5-benzyl-5H-pyrrolo[3,2-d]pyrimidine* (**9**). ^1^H-NMR (400 MHz, CDCl_3_) (ppm) δ: 5.67 (s, 2H); 6.71 (d, *J* = 3.2 Hz, 1H); 7.05 (d, *J* = 6.44 Hz, 2H); 7.34 (m, 3H); 7.53 (d, *J* = 3.2 Hz, 1H). ^13^C-NMR (100 MHz, CDCl_3_): 154.4,4, 150.61, 143.16, 138.92, 136.36, 129.25, 128.48, 126.63, 123.15, 103.00, 52.41. ESI-MS *m*/*z* for C_13_H_9_Cl_2_N_3_ calcd. at 277.02, found at 278.03 [M + H]^+^. Anal. calcd. for C_13_H_9_Cl_2_N_3_: C, 56.14; H, 3.26; N, 15.11. Found: C, 56.11; H, 3.26; N, 15.10.

*2,4-dichloro-5-(2,4-dichlorobenzyl)-5H-pyrrolo[3,2-d]pyrimidine* (**10**). ^1^H-NMR (400 MHz, CDCl_3_) (ppm) δ: 5.72 (s, 2H); 6.42 (d, *J* = 8.68 Hz, 1H); 6.75 (d, *J* = 3.24 Hz, 1H); 7.15 (d, *J* = 8.28 Hz, 1H); 7.46 (d, *J* = 2.28 Hz, 1H); 7.51 (d *J* = 3.20 Hz, 1H). ^13^C-NMR (100 MHz, DMSO-*d*_6_): δ154.39, 149.55, 142.77, 142.23, 135.14, 133.24, 132.01, 129.52, 128.13, 122.50, 103.03, 49.67. ESI-MS *m*/*z* for C_13_H_7_Cl_4_N_3_ calcd. at 344.94, found at 345.95 [M + H]^+^. Anal. calcd. for C_13_H_7_Cl_4_N_3_: C, 45.00; H, 2.03; N, 12.11. Found: C, 44.96; H, 2.03; N, 12.09.

*2,4-dichloro-5-(4-methoxybenzyl)-5H-pyrrolo[3,2-d]pyrimidine* (**11**). ^1^H-NMR (400 MHz, CDCl_3_) (ppm) δ: 3.78 (t, *J* = 7.36 Hz, 3H); 5.60 (d, *J* = 3.68 Hz, 2H); 6.67 (s, 1H); 6.86 (q, *J* = 4.12 Hz, 2H); 7.03 (t, *J* = 3.68 Hz, 2H); 7.50 (s, 1H). ^13^C-NMR (100 MHz, DMSO-*d*_6_): δ159.48, 155.10, 149.01, 142.96, 141.49, 141.00, 130.06, 128.35, 123.01, 114.97, 102.07, 55.61, 51.20. ESI-MS *m*/*z* for C_14_H_11_Cl_2_N_3_O calcd. at 307.03, found at 307.93 [M + H]^+^. Anal. calcd. for C_14_H_11_C_l2_N_3_O: C, 54.57; H, 3.60; N, 13.64. Found: C, 54.54; H, 3.60; N, 13.63.

*1-(2,4-dichloro-5H-pyrrolo[3,2-d]pyrimidin-5-yl)ethan-1-one* (**12**). ^1^H-NMR (400 MHz, CDCl_3_) (ppm) δ: 2.73 (s, 3H); 6.77 (d, *J* = 3.64 Hz, 1H); 7.92 (d, *J* = 4.12 Hz, 1H). ^13^C-NMR (100 MHz, DMSO-*d*_6_): δ168.26, 158.29, 152.17, 145.87, 140.28, 123.01, 107.93, 24.18. ESI-MS *m*/*z* for C_8_H_5_Cl_2_N_3_O calcd. at 228.98, found at 229.54 [M + H]^+^. Anal. calcd. for C_8_H_5_Cl_2_N_3_O: C, 41.77; H, 12.19; N, 18.27. Found: C, 41.75; H, 2.19; N, 18.25.

*1-(2,4-dichloro-5H-pyrrolo[3,2-d]pyrimidin-5-yl)-2-methylpropan-1-one* (**13**). ^1^H-NMR (400 MHz, CDCl_3_) (ppm) δ: 1.37 (d, *J* = 6.84 Hz, 6H); 3.33 (septet, *J* = 6.88 Hz, 1H); 6.77 (d, *J* = 3.68 Hz, 1H); 7.92 (d, *J* = 3.64 Hz, 1H). ^13^C-NMR (100 MHz, DMSO-*d*_6_): δ174.60, 158.78, 152.44, 146.37, 138.82, 124.21, 106.45, 34.17, 19.07. ESI-MS *m*/*z* for C_10_H_9_Cl_2_N=O calcd. at 257.01, found at 257.68 [M + H]^+^. Anal. calcd. for C_10_H_9_Cl_2_N_3_O: C, 46.54; H, 3.51; N, 16.28. Found: C, 46.51; H, 3.51; N, 16.27.

*2,4-dichloro-N,N-diphenyl-5H-pyrrolo[3,2-d]pyrimidine-5-carboxamide* (**14**). ^1^H-NMR (400 MHz, DMSO-*d*_6_) (ppm) δ: 6.76 (d, *J* = 3.20 Hz, 1H); 7.23–7.34 (m, 10H); 8.37 (d, *J* = 3.20 Hz, 1H). ^13^C-NMR (100 MHz, DMSO-*d*_6_): δ155.30, 151.12, 149.65, 144.15, 141.73, 139.55, 130.09, 123.10, 104.63. ESI-MS *m*/*z* for C_19_H_12_Cl_2_N_4_O calcd. at 382.04, found at 383.04 [M + H]^+^. Anal. calcd. for C_19_H_12_Cl_2_N_4_O: C, 59.55; H, 3.16; N, 14.62. Found: C, 59.53; H, 3.16; N, 14.61.

*2-(2,4-dichloro-5H-pyrrolo[3,2-d]pyrimidin-5-yl)ethan-1-ol* (**15**). ^1^H-NMR (400 MHz, DMSO-*d*_6_) (ppm) δ: 3.70 (d, *J* = 5.52 Hz, 2H); 4.50 (t, *J* = 5.48 Hz, 2H); 6.68 (d, *J* = 3.24 Hz, 1H); 8.05 (d, *J* = 3.20 Hz, 1H); 8.08 (s, 1H). ^13^C-NMR (100 MHz, DMSO-*d*_6_): δ153.40, 142.99, 137.59, 124.25, 102.56, 101.54, 61.40, 51.47. ESI-MS *m*/*z* for C_8_H_7_Cl_2_N_3_O calcd. at 231.00, found at 231.93 [M + H]^+^

## Figures and Tables

**Table 1:** Best Growth Percent and Target Cell Line for Compounds **5**–**15.**

Compound	Best Growth Percent *	Most Affected Cell Line
**5**	60.77	LOX-IMVI (Melanoma)
**6**	84.49	NCI-H522; NSC Lung
**7**	49.57	NCI-H522; NSC Lung
**8**	93.16	UO-31; Renal
**9**	36.31	CCRF-CEM; Leukemia
**10**	22.88	CCRF-CEM; Leukemia
**11**	65.83	CCRF-CEM; Leukemia
**12**	81.36	UO-31; Renal
**13**	75.18	SR, Leukemia
**14**	−0.95	MDA-MB-468; Breast
**15**	89.73	NCI-H522; NSC Lung

* Represents the growth percent in the most affected cell line (i.e., highest antiproliferative activity exhibited in the NCI-60 cell screening).

**Table 2:** Pearson Correlation Coefficient of Selected Compounds with Target Vector and Common Cell Line Count.

Compound	Correlation (PCC)	Target Vector ^a^	Common Cell Line Count ^b^
**9**	0.717	D-Tetrandrine	55
**7**	0.661	Pibenzimol HCl	57
**9**	0.640	Fluorodopan	58
**9**	0.622	Melphalan	58
**9**	0.622	Carmustine (BCNU)	59

^a^ Known compound with activity most comparable to experimental compound; ^b^ Number of cell lines displaying similar activity between experimental compound and target vector.

**Table 3:** Physical and pharmacokinetic properties of active pyrrolo[3,2-*d*]pyrimidines.

Compound	SASA ^a^ (Å^2^)	EA ^b^ (eV)	QPPCaco ^c^ (nm/sec)	QPlogS ^d^ (log[mol/dm^3^])
**7**	514.262	9.613	243.855	−2.017
**9**	476.642	8.878	3989.857	−4.405
**10**	509.662	8.947	4018.835	−5.547
**14**	601.2	9.071	1349.875	−4.892

^a^ solvent accessible surface area; ^b^ electron affinity; ^c^ predicted apparent Caco-2 cell permeability; ^d^ predicted aqueous solubility.

## Supplementary Materials

The following are available online, Figure S1: Complete NCI-60 Mean Growth Charts, Figure S2: ^1^H-NMR of Compounds **6**–**15**, Figure S3: ^13^C-NMR of Compounds **6**–**15.**
