# Supplementary material for: Structural and Biological Investigations for a Series of N-5 Substituted Pyrrolo[3,2-d]pyrimidines as Potential Anti-Cancer Therapeutics
Source: Molecules. 2019 Jul 23;24(14):2656. doi: 10.3390/molecules24142656 (PMC6680647; doi:10.3390/molecules24142656)
Supplement: Supplementary file 1 [file molecules-24-02656-s001.pdf]

Supplemental Information

Structural and Biological Investigations for a Series of N-5 Substituted Pyrrolo[3,2-*d*]pyrimidines as Potential Anticancer Therapeutics

Brian M. Cawrse, Nia'Mani M. Robinson, Nina C. Lee, Gerald M. Wilson, Katherine L. Seley-Radtke

<u>Table of Contents</u>

Figure S1: NCI-60 Human Cancer Cell Line Mean Growth Charts **2-11**

Figure S2: ^{1}H NMR Spectra **12-21**

Figure S3: ^{13}C NMR Spectra **22-31**

Figure S1. Figure S1. NCI-60 Human Cancer Cell Line Mean Growth Charts

Developmental Therapeutics Program One Dose Mean Graph	NSC: D-807630 / 1	Conc: 1.00E-5 Molar	Test Date: Sep 17, 2018
	Experiment ID: 1809OS05		Report Date: Nov 05, 2018

Panel/Cell Line	Growth Percent
Leukemia	
CCRF-CEM	94.89
HL-60(TB)	100.62
K-562	98.99
MOLT-4	100.64
RPMI-8226	99.42
SR	85.91
Non-Small Cell Lung Cancer	
A549/ATCC	102.68
EKVX	100.72
HOP-62	101.55
HOP-92	107.69
NCI-H226	100.72
NCI-H23	100.45
NCI-H322M	104.03
NCI-H460	103.52
NCI-H522	84.49
Colon Cancer	
COLO 205	109.64
HCC-2998	105.74
HCT-116	100.88
HCT-15	97.43
HT29	99.85
KM12	102.97
SW-620	106.62
CNS Cancer	
SF-268	100.41
SF-295	101.12
SF-539	100.78
SNB-19	101.18
SNB-75	99.33
U251	93.58
Melanoma	
LOX IMVI	96.10
MALME-3M	94.04
M14	94.65
MDA-MB-435	101.89
SK-MEL-2	101.75
SK-MEL-28	104.86
SK-MEL-5	99.65
UACC-257	97.36
UACC-62	101.35
Ovarian Cancer	
IGROV1	93.20
OVCAR-3	114.13
OVCAR-4	106.44
OVCAR-5	105.46
OVCAR-8	104.83
NCI/ADR-RES	108.14
SK-OV-3	103.55
Renal Cancer	
786-0	101.56
A498	114.23
ACHN	107.19
CAKI-1	104.12
RXF 393	100.54
SN12C	99.55
TK-10	112.22
UO-31	96.85
Prostate Cancer	
DU-145	109.69
Breast Cancer	
MCF7	85.39
MDA-MB-231/ATCC	109.33
HS 578T	103.51
BT-549	97.41
T-47D	94.67
MDA-MB-468	94.30
Mean	101.08
Delta	16.59
Range	29.74

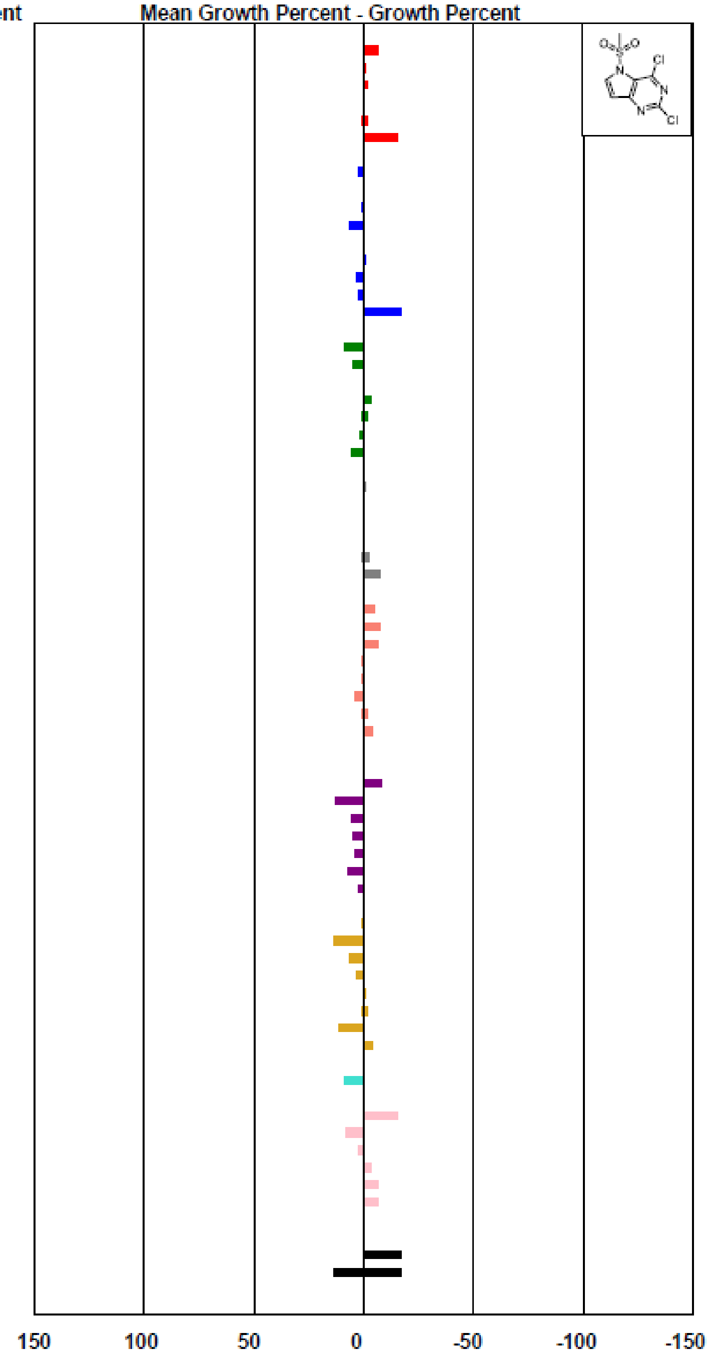

Developmental Therapeutics Program One Dose Mean Graph	NSC: D-807631 / 1	Conc: 1.00E-5 Molar	Test Date: Sep 17, 2018
	Experiment ID: 1809OS05		Report Date: Nov 05, 2018

Panel/Cell Line	Growth Percent
**Leukemia**	
CCRF-CEM	59.91
HL-60(TB)	87.46
K-562	69.67
MOLT-4	89.33
RPMI-8226	90.25
SR	61.36
**Non-Small Cell Lung Cancer**	
A549/ATCC	97.77
EKVX	96.63
HOP-62	97.90
HOP-92	97.11
NCI-H226	84.37
NCI-H23	93.79
NCI-H322M	100.03
NCI-H460	101.07
NCI-H522	49.57
**Colon Cancer**	
COLO 205	108.32
HCC-2998	108.30
HCT-116	81.41
HCT-15	84.23
HT29	96.53
KM12	95.47
SW-620	91.96
**CNS Cancer**	
SF-268	94.05
SF-295	105.01
SF-539	100.31
SNB-19	98.97
SNB-75	87.67
U251	92.02
**Melanoma**	
LOX IMVI	83.31
MALME-3M	86.48
M14	87.63
MDA-MB-435	92.36
SK-MEL-2	90.40
SK-MEL-28	101.18
SK-MEL-5	94.89
UACC-257	94.42
UACC-62	93.53
**Ovarian Cancer**	
IGROV1	83.59
OVCAR-3	103.89
OVCAR-4	91.44
OVCAR-5	112.38
OVCAR-8	94.54
NCI/ADR-RES	104.57
SK-OV-3	93.94
**Renal Cancer**	
786-0	103.81
A498	124.33
ACHN	99.18
CAKI-1	93.39
RXF 393	100.07
SN12C	93.59
TK-10	117.26
UO-31	88.72
**Prostate Cancer**	
DU-145	104.74
**Breast Cancer**	
MCF7	79.05
MDA-MB-231/ATCC	105.62
HS 578T	96.86
BT-549	99.22
T-47D	77.15
MDA-MB-468	76.08
Mean	93.02
Delta	43.45
Range	74.76

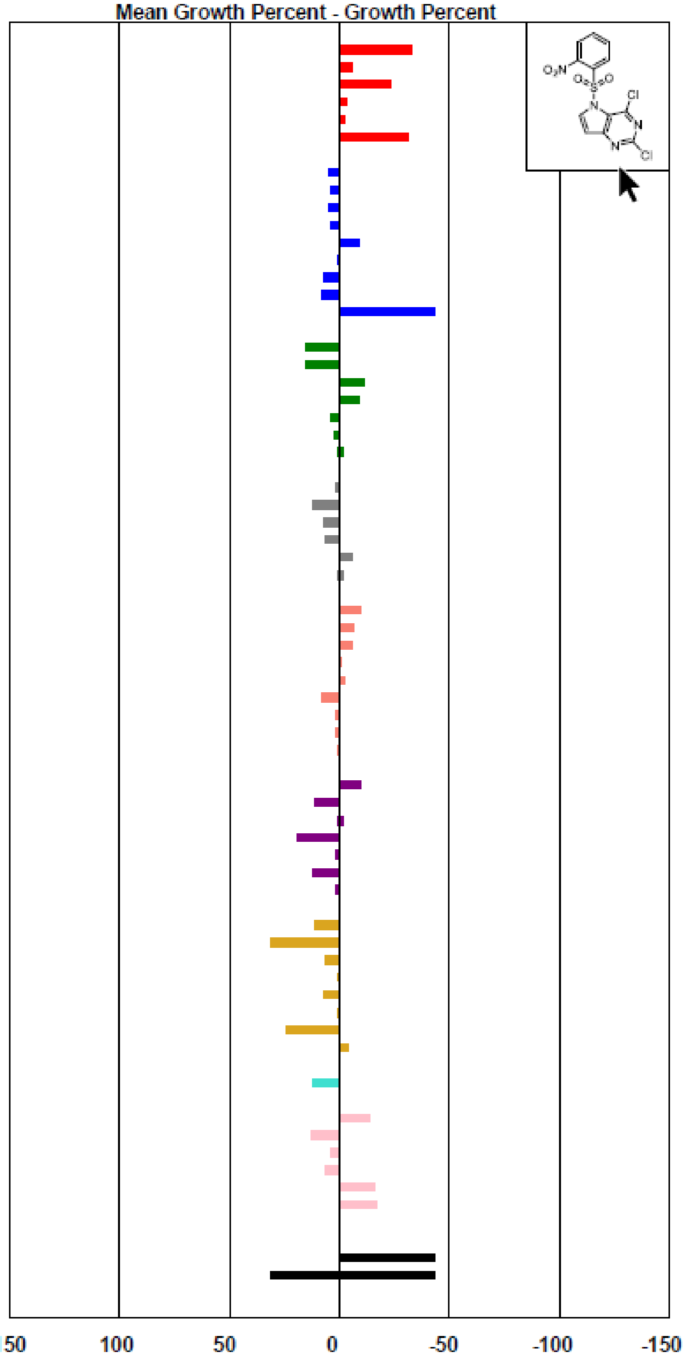

**Developmental Therapeutics Program**<br>**One Dose Mean Graph**	**NSC:** D-807632 / 1	**Conc:** 1.00E-5 Molar	**Test Date:** Sep 17, 2018
	**Experiment ID:** 1809OS05		**Report Date:** Nov 05, 2018

Panel/Cell Line	Growth Percent
Leukemia	
CCRF-CEM	104.04
HL-60(TB)	98.09
K-562	103.77
MOLT-4	98.33
RPMI-8226	110.66
SR	108.84
Non-Small Cell Lung Cancer	
A549/ATCC	102.51
EKVX	108.17
HOP-62	108.83
HOP-92	99.90
NCI-H226	98.17
NCI-H23	98.30
NCI-H322M	104.62
NCI-H460	103.31
NCI-H522	96.14
Colon Cancer	
COLO 205	109.88
HCC-2998	102.06
HCT-116	97.75
HCT-15	102.36
HT29	112.60
KM12	106.39
SW-620	106.52
CNS Cancer	
SF-268	97.82
SF-295	104.27
SF-539	98.17
SNB-19	99.09
SNB-75	94.91
U251	94.04
Melanoma	
LOX IMVI	96.60
MALME-3M	102.76
M14	105.92
MDA-MB-435	103.86
SK-MEL-2	102.67
SK-MEL-28	108.98
SK-MEL-5	102.25
UACC-257	104.89
UACC-62	101.22
Ovarian Cancer	
IGROV1	100.15
OVCAR-3	108.56
OVCAR-4	104.98
OVCAR-5	104.99
OVCAR-8	102.10
NCI/ADR-RES	103.48
SK-OV-3	105.95
Renal Cancer	
786-0	105.17
A498	113.29
ACHN	104.40
CAKI-1	97.02
RXF 393	117.43
SN12C	97.53
TK-10	103.23
UO-31	93.16
Prostate Cancer	
DU-145	112.03
Breast Cancer	
MCF7	98.76
MDA-MB-231/ATCC	105.13
HS 578T	104.15
BT-549	99.51
T-47D	104.08
MDA-MB-468	121.13
Mean	103.47
Delta	10.31
Range	27.97

Mean Growth Percent - Growth Percent

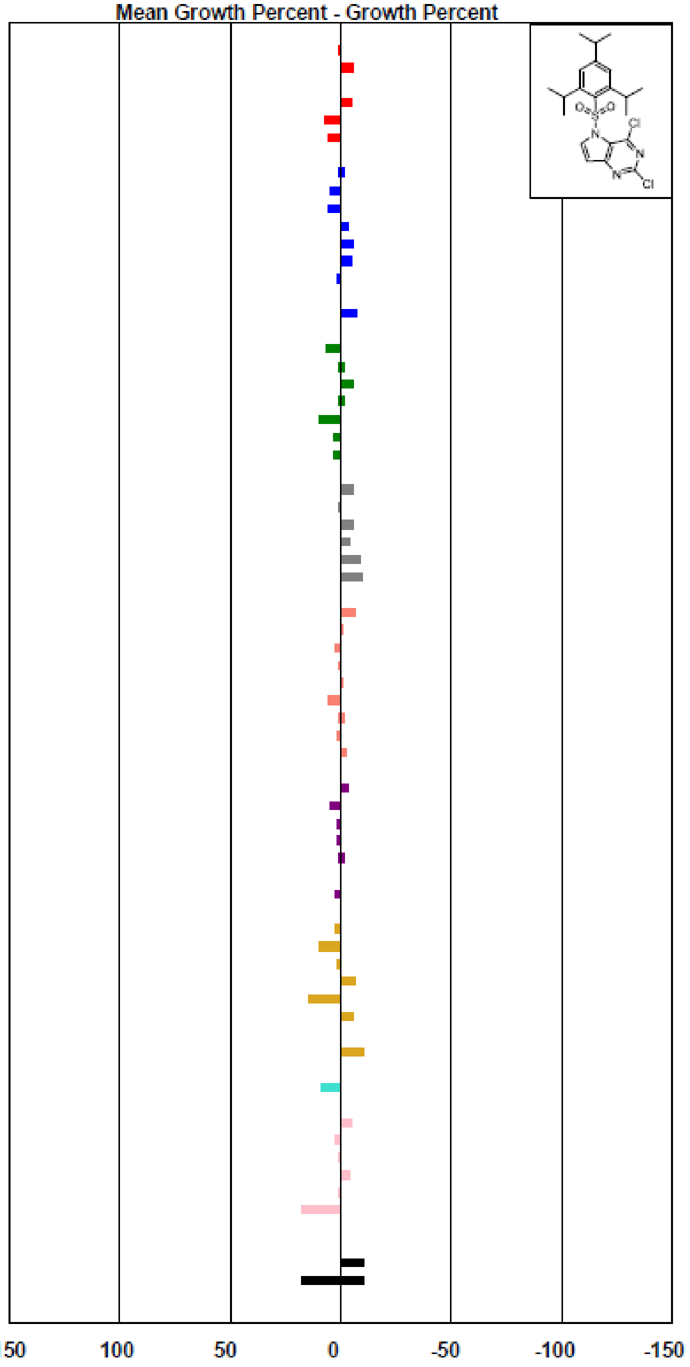

Developmental Therapeutics Program	NSC: D-807633 / 1	Conc: 1.00E-5 Molar	Test Date: Sep 17, 2018
One Dose Mean Graph	Experiment ID: 1809OS05		Report Date: Nov 05, 2018

Panel/Cell Line	Growth Percent
Leukemia	
CCRF-CEM	36.31
HL-60(TB)	77.87
K-562	72.55
MOLT-4	56.49
RPMI-8226	85.87
SR	37.66
Non-Small Cell Lung Cancer	
A549/ATCC	100.16
EKVX	96.92
HOP-62	104.68
HOP-92	85.99
NCI-H226	91.98
NCI-H23	92.95
NCI-H322M	106.21
NCI-H460	103.03
NCI-H522	84.90
Colon Cancer	
COLO 205	103.71
HCC-2998	103.07
HCT-116	78.11
HCT-15	95.10
HT29	94.99
KM12	92.33
SW-620	59.77
CNS Cancer	
SF-268	96.32
SF-295	101.42
SF-539	87.17
SNB-19	92.58
SNB-75	95.55
U251	83.24
Melanoma	
LOX IMVI	88.48
MALME-3M	85.33
M14	80.36
MDA-MB-435	77.82
SK-MEL-2	98.23
SK-MEL-28	105.53
SK-MEL-5	98.45
UACC-257	92.88
UACC-62	94.89
Ovarian Cancer	
IGROV1	96.12
OVCAR-3	107.93
OVCAR-4	107.18
OVCAR-5	103.07
OVCAR-8	96.53
NCI/ADR-RES	102.90
SK-OV-3	96.83
Renal Cancer	
786-0	100.91
A498	116.70
ACHN	93.61
CAKI-1	97.50
RXF 393	108.59
SN12C	69.34
TK-10	109.54
UO-31	84.74
Prostate Cancer	
DU-145	106.91
Breast Cancer	
MCF7	69.33
MDA-MB-231/ATCC	90.12
HS 578T	97.23
BT-549	100.67
T-47D	94.33
MDA-MB-468	106.99
Mean	91.46
Delta	55.15
Range	80.39

Mean Growth Percent - Growth Percent

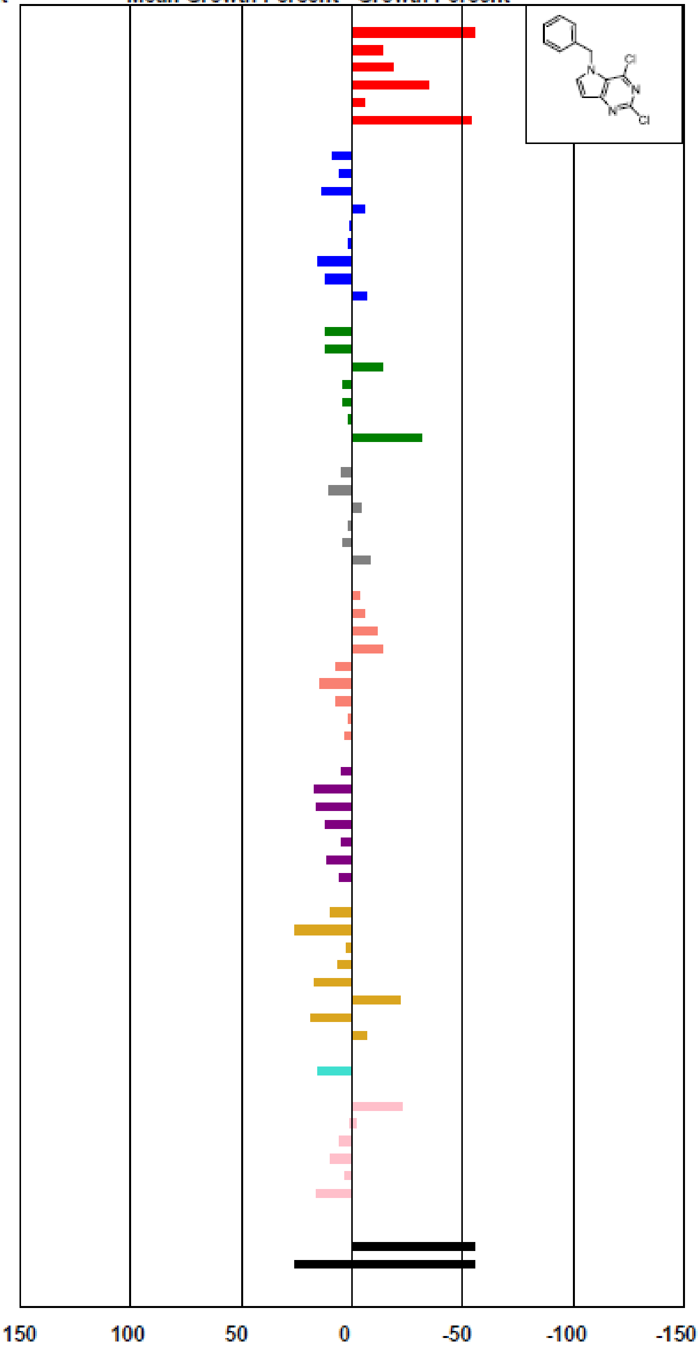

Developmental Therapeutics Program<br>One Dose Mean Graph	NSC: D-807634 / 1	Conc: 1.00E-5 Molar	Test Date: Sep 17, 2018
	Experiment ID: 1809OS05		Report Date: Nov 05, 2018

Panel/Cell Line	Growth Percent
Leukemia	
CCRF-CEM	22.88
HL-60(TB)	90.78
K-562	51.26
MOLT-4	36.43
RPMI-8226	66.58
SR	45.32
Non-Small Cell Lung Cancer	
A549/ATCC	97.79
EKVX	99.04
HOP-62	100.20
HOP-92	113.57
NCI-H226	61.01
NCI-H23	63.50
NCI-H322M	96.96
NCI-H460	99.13
NCI-H522	35.12
Colon Cancer	
COLO 205	114.15
HCC-2998	101.41
HCT-116	56.68
HCT-15	78.46
HT29	92.69
KM12	97.64
SW-620	61.84
CNS Cancer	
SF-268	91.77
SF-295	104.66
SF-539	105.26
SNB-19	96.26
SNB-75	100.90
U251	101.81
Melanoma	
LOX IMVI	79.32
MALME-3M	85.42
M14	81.94
MDA-MB-435	70.69
SK-MEL-2	86.11
SK-MEL-28	101.70
SK-MEL-5	81.57
UACC-257	98.44
UACC-62	86.82
Ovarian Cancer	
IGROV1	96.92
OVCAR-3	99.16
OVCAR-4	91.70
OVCAR-5	119.01
OVCAR-8	75.17
NCI/ADR-RES	82.01
SK-OV-3	98.74
Renal Cancer	
786-0	105.70
A498	121.24
ACHN	102.72
CAKI-1	119.41
RXF 393	104.11
SN12C	83.45
TK-10	108.44
UO-31	105.59
Prostate Cancer	
DU-145	77.17
Breast Cancer	
MCF7	75.52
MDA-MB-231/ATCC	107.67
HS 578T	90.10
BT-549	74.39
T-47D	66.07
MDA-MB-468	43.59
Mean	86.49
Delta	63.61
Range	98.36

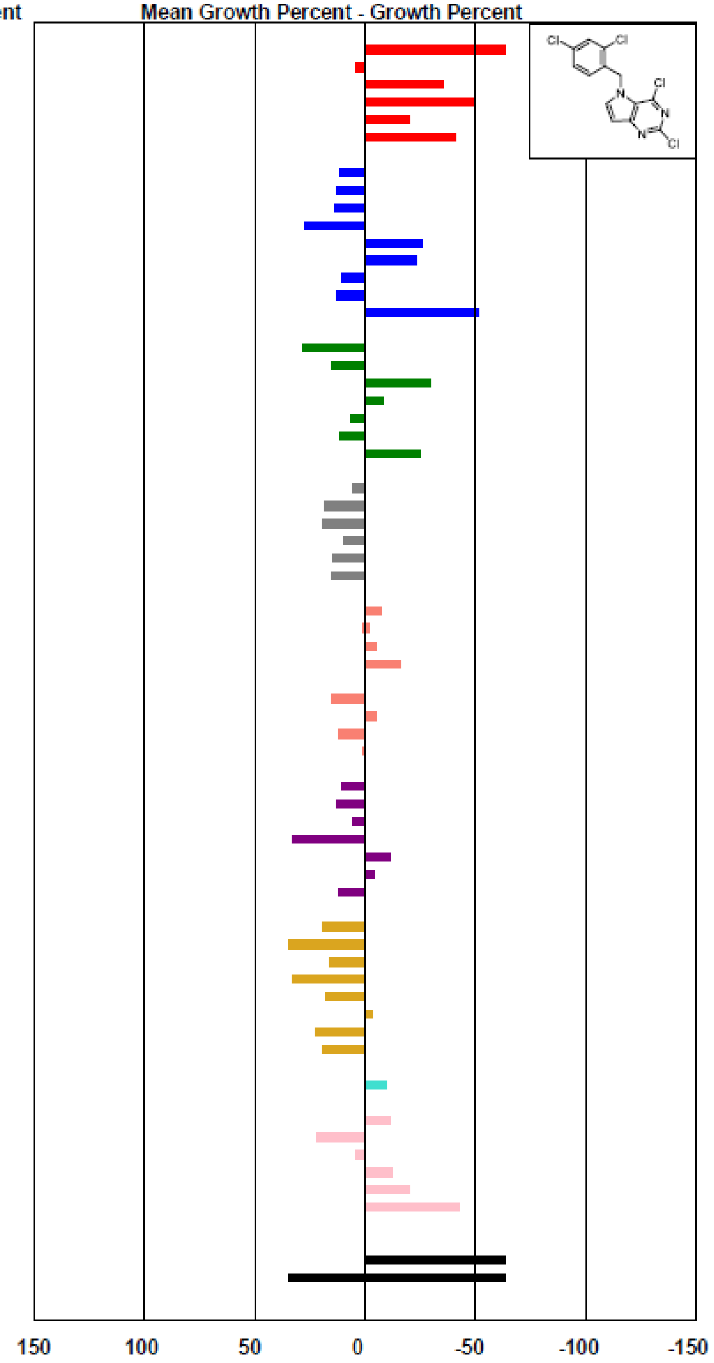

Developmental Therapeutics Program One Dose Mean Graph	NSC: D-807635 / 1	Conc: 1.00E-5 Molar	Test Date: Sep 17, 2018
	Experiment ID: 1809OS05		Report Date: Nov 05, 2018

Panel/Cell Line	Growth Percent
**Leukemia**	
CCRF-CEM	65.83
HL-60(TB)	98.04
K-562	83.29
MOLT-4	76.58
RPMI-8226	92.75
SR	82.82
**Non-Small Cell Lung Cancer**	
A549/ATCC	103.46
EKVX	99.28
HOP-62	98.94
HOP-92	100.59
NCI-H226	84.89
NCI-H23	87.42
NCI-H322M	97.77
NCI-H460	101.94
NCI-H522	70.40
**Colon Cancer**	
COLO 205	111.57
HCC-2998	105.41
HCT-116	94.49
HCT-15	99.28
HT29	108.54
KM12	102.16
SW-620	108.44
**CNS Cancer**	
SF-268	102.91
SF-295	104.33
SF-539	100.42
SNB-19	100.48
SNB-75	96.49
U251	98.86
**Melanoma**	
LOX IMVI	87.11
MALME-3M	83.17
M14	90.98
MDA-MB-435	99.20
SK-MEL-2	97.89
SK-MEL-28	106.20
SK-MEL-5	87.70
UACC-257	101.32
UACC-62	93.28
**Ovarian Cancer**	
IGROV1	105.97
OVCAR-3	108.20
OVCAR-4	93.22
OVCAR-5	107.28
OVCAR-8	96.74
NCI/ADR-RES	104.42
SK-OV-3	102.07
**Renal Cancer**	
786-0	106.49
A498	111.58
ACHN	104.62
CAKI-1	106.30
RXF 393	117.38
SN12C	93.44
TK-10	103.42
UO-31	94.91
**Prostate Cancer**	
DU-145	93.66
**Breast Cancer**	
MCF7	93.57
MDA-MB-231/ATCC	106.32
HS 578T	94.34
BT-549	95.26
T-47D	77.46
MDA-MB-468	60.21
Mean	96.63
Delta	36.42
Range	57.17

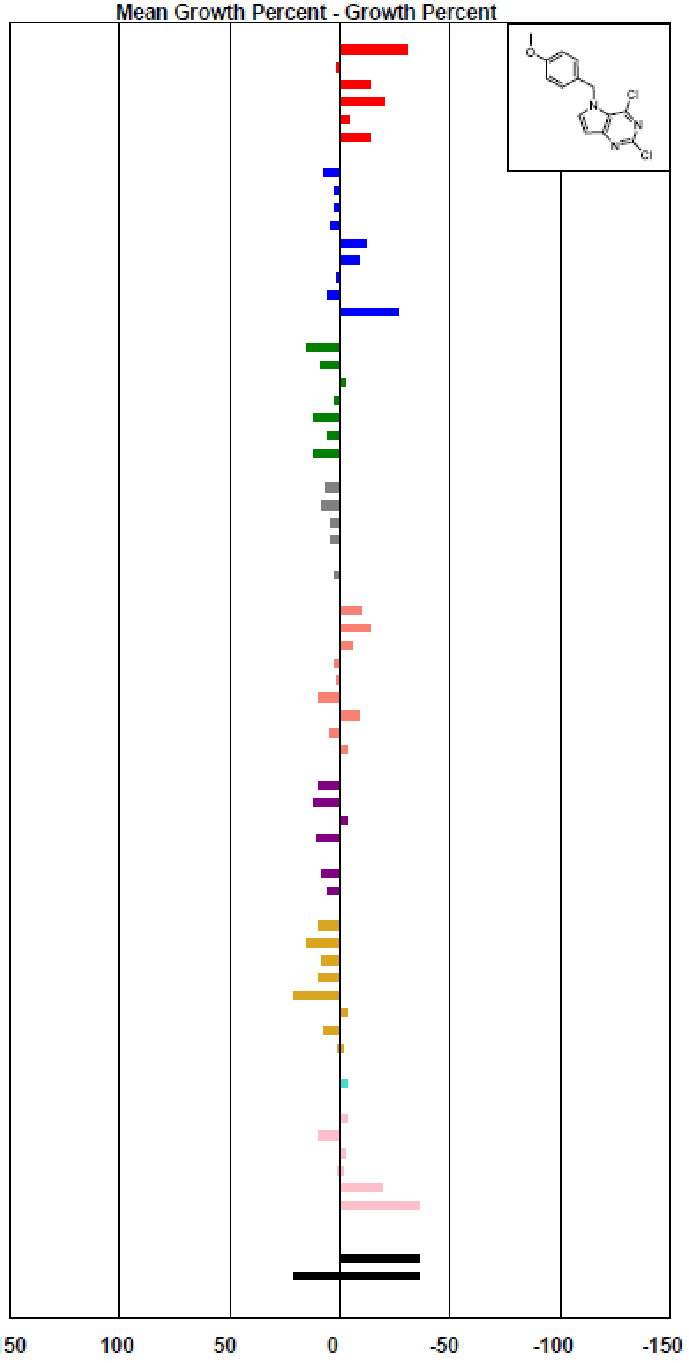

Developmental Therapeutics Program One Dose Mean Graph	NSC: D-807636 / 1	Conc: 1.00E-5 Molar	Test Date: Sep 17, 2018
	Experiment ID: 1809OS05		Report Date: Nov 05, 2018

Panel/Cell Line	Growth Percent
**Leukemia**	
CCRF-CEM	107.29
HL-60(TB)	99.96
K-562	97.27
MOLT-4	106.03
RPMI-8226	103.61
SR	87.34
**Non-Small Cell Lung Cancer**	
A549/ATCC	102.07
EKVX	93.88
HOP-62	96.77
HOP-92	95.68
NCI-H226	92.55
NCI-H23	89.06
NCI-H322M	94.70
NCI-H460	102.16
NCI-H522	89.40
**Colon Cancer**	
COLO 205	106.87
HCC-2998	101.66
HCT-116	106.98
HCT-15	103.51
HT29	107.65
KM12	94.57
SW-620	99.48
**CNS Cancer**	
SF-268	97.92
SF-295	100.23
SF-539	92.51
SNB-19	99.98
SNB-75	88.50
U251	86.97
**Melanoma**	
LOX IMVI	93.51
MALME-3M	88.67
M14	101.57
MDA-MB-435	99.79
SK-MEL-2	99.89
SK-MEL-28	103.84
SK-MEL-5	101.29
UACC-257	92.55
UACC-62	95.43
**Ovarian Cancer**	
IGROV1	96.96
OVCAR-3	106.92
OVCAR-4	100.79
OVCAR-5	106.45
OVCAR-8	97.76
NCI/ADR-RES	100.39
SK-OV-3	96.04
**Renal Cancer**	
786-0	105.16
A498	121.00
ACHN	98.79
CAKI-1	87.19
RXF 393	107.83
SN12C	96.96
TK-10	115.50
UO-31	81.36
**Prostate Cancer**	
DU-145	100.59
**Breast Cancer**	
MCF7	88.05
MDA-MB-231/ATCC	102.26
HS 578T	93.46
BT-549	98.53
T-47D	88.07
MDA-MB-468	110.62
Mean	98.67
Delta	17.31
Range	39.64

Mean Growth Percent - Growth Percent

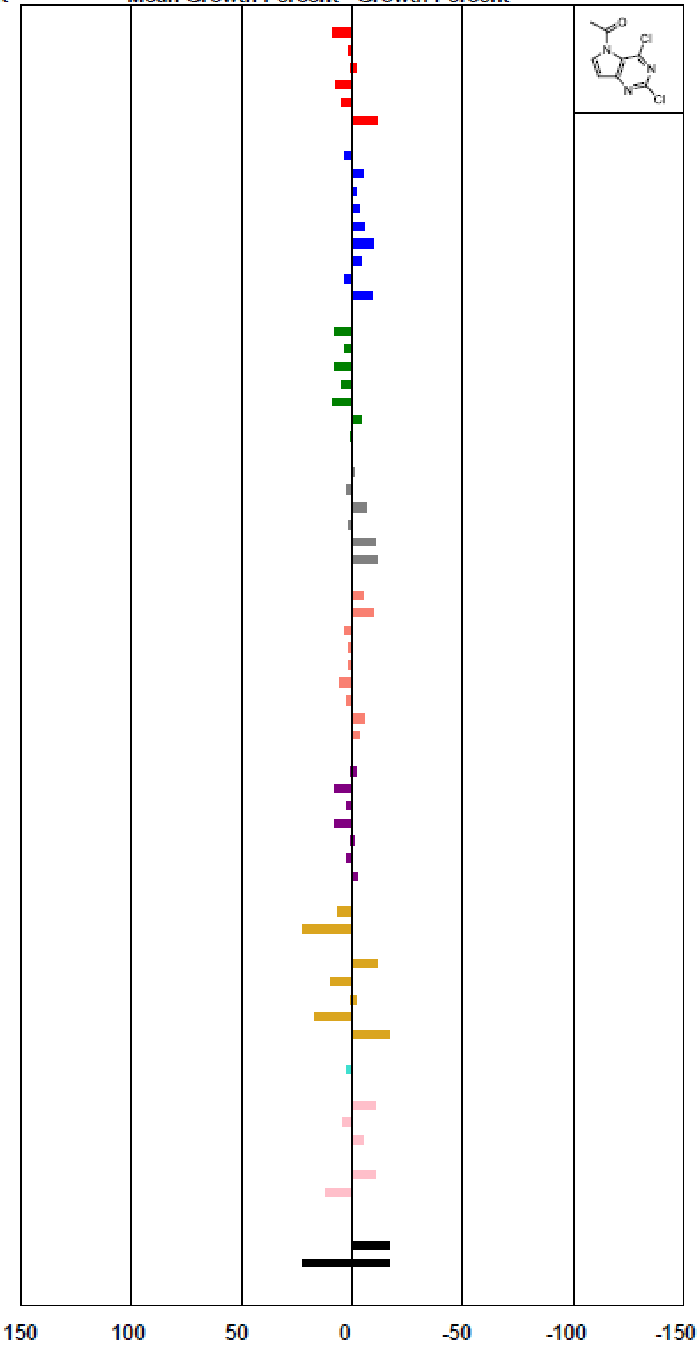

Developmental Therapeutics Program One Dose Mean Graph	NSC: D-807637 / 1	Conc: 1.00E-5 Molar	Test Date: Sep 17, 2018
	Experiment ID: 1809OS05		Report Date: Nov 05, 2018

Panel/Cell Line	Growth Percent
**Leukemia**	
CCRF-CEM	99.27
HL-60(TB)	98.80
K-562	96.01
MOLT-4	103.50
RPMI-8226	100.34
SR	75.18
**Non-Small Cell Lung Cancer**	
A549/ATCC	101.53
EKVX	92.17
HOP-62	99.14
HOP-92	90.00
NCI-H226	94.59
NCI-H23	96.96
NCI-H322M	95.83
NCI-H460	102.13
NCI-H522	89.32
**Colon Cancer**	
COLO 205	116.68
HCC-2998	101.02
HCT-116	98.24
HCT-15	98.56
HT29	106.30
KM12	96.38
SW-620	101.48
**CNS Cancer**	
SF-268	98.38
SF-295	99.73
SF-539	93.67
SNB-19	98.84
SNB-75	90.68
U251	84.84
**Melanoma**	
LOX IMVI	93.42
MALME-3M	88.62
M14	96.56
MDA-MB-435	96.84
SK-MEL-2	98.27
SK-MEL-28	101.53
SK-MEL-5	99.72
UACC-257	102.89
UACC-62	101.72
**Ovarian Cancer**	
IGROV1	101.27
OVCAR-3	107.49
OVCAR-4	91.91
OVCAR-5	107.03
OVCAR-8	101.78
NCI/ADR-RES	103.99
SK-OV-3	95.52
**Renal Cancer**	
786-0	101.38
A498	115.95
ACHN	97.47
CAKI-1	94.61
RXF 393	97.31
SN12C	98.53
TK-10	104.06
UO-31	88.38
**Prostate Cancer**	
DU-145	98.89
**Breast Cancer**	
MCF7	86.51
MDA-MB-231/ATCC	96.53
HS 578T	96.31
BT-549	97.72
T-47D	85.26
MDA-MB-468	107.55
Mean	97.87
Delta	22.69
Range	41.50

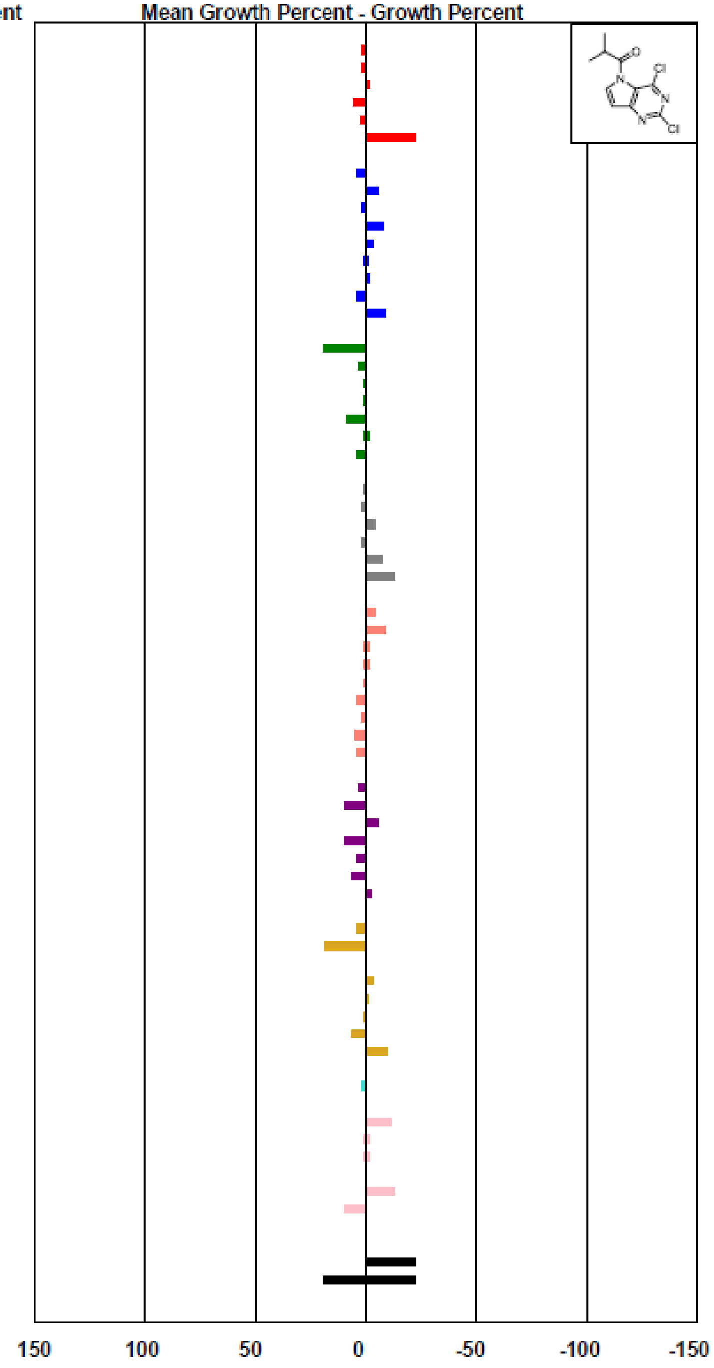

Developmental Therapeutics Program<br>One Dose Mean Graph	NSC: D-807638 / 1	Conc: 1.00E-5 Molar	Test Date: Sep 17, 2018
	Experiment ID: 1809OS05		Report Date: Nov 05, 2018

Panel/Cell Line	Growth Percent
Leukemia	
CCRF-CEM	15.98
HL-60(TB)	49.50
K-562	42.83
MOLT-4	37.13
RPMI-8226	16.10
SR	54.36
Non-Small Cell Lung Cancer	
A549/ATCC	56.19
EKVX	63.28
HOP-62	45.45
HOP-92	52.30
NCI-H226	53.88
NCI-H23	60.79
NCI-H322M	67.89
NCI-H460	36.14
NCI-H522	24.88
Colon Cancer	
COLO 205	77.71
HCC-2998	91.55
HCT-116	49.58
HCT-15	45.11
HT29	24.64
KM12	55.66
SW-620	51.07
CNS Cancer	
SF-268	66.26
SF-295	74.07
SF-539	68.00
SNB-19	58.48
SNB-75	38.29
U251	59.63
Melanoma	
LOX IMVI	72.12
MALME-3M	58.65
M14	69.91
MDA-MB-435	37.92
SK-MEL-2	58.55
SK-MEL-28	80.42
SK-MEL-5	64.89
UACC-257	70.15
UACC-62	51.93
Ovarian Cancer	
IGROV1	68.47
OVCAR-3	30.05
OVCAR-4	63.29
OVCAR-5	64.22
OVCAR-8	63.17
NCI/ADR-RES	35.81
SK-OV-3	49.73
Renal Cancer	
786-0	80.09
A498	80.98
ACHN	65.24
CAKI-1	53.41
RXF 393	32.74
SN12C	53.81
TK-10	73.67
UO-31	57.13
Prostate Cancer	
DU-145	33.82
Breast Cancer	
MCF7	32.54
MDA-MB-231/ATCC	71.37
HS 578T	42.24
BT-549	47.20
T-47D	34.21
MDA-MB-468	-0.95
Mean	53.11
Delta	54.06
Range	92.50

Mean Growth Percent - Growth Percent

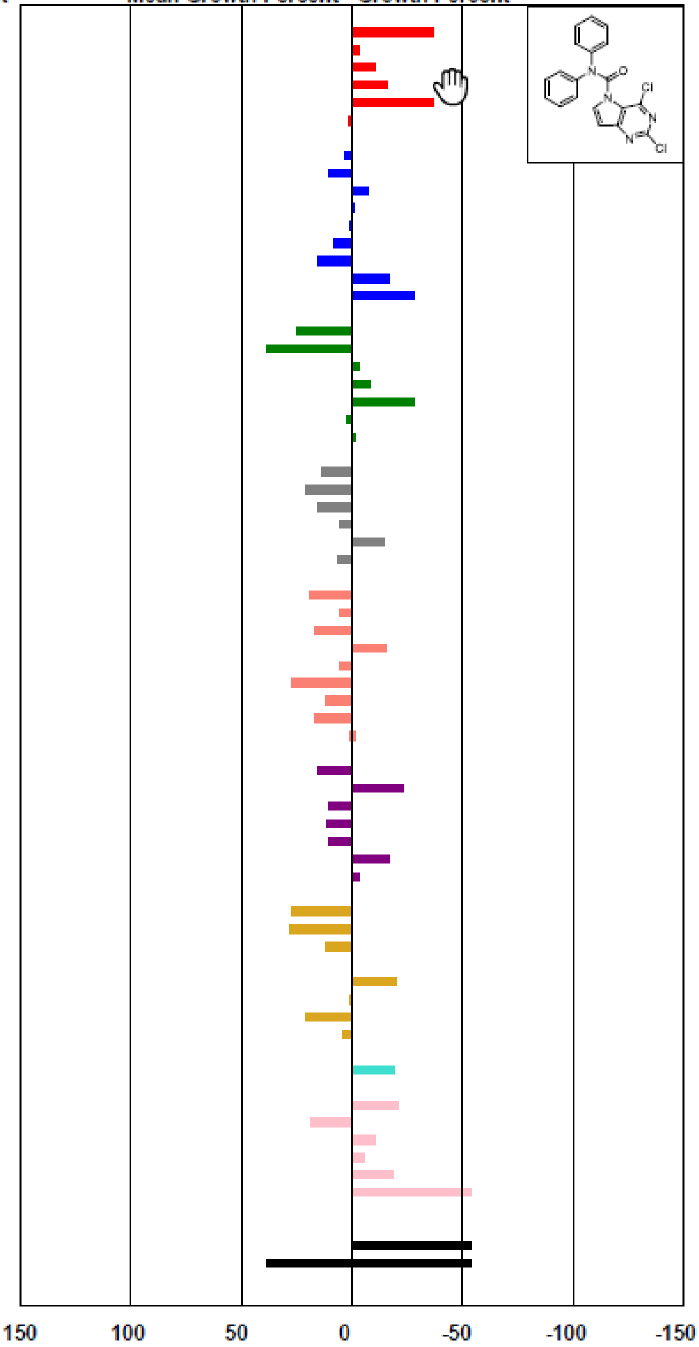

Developmental Therapeutics Program One Dose Mean Graph	NSC: D-807639 / 1	Conc: 1.00E-5 Molar	Test Date: Sep 17, 2018
	Experiment ID: 1809OS05		Report Date: Nov 05, 2018

Panel/Cell Line	Growth Percent
**Leukemia**	
CCRF-CEM	98.95
HL-60(TB)	99.35
K-562	103.70
MOLT-4	102.98
RPMI-8226	101.39
SR	92.76
**Non-Small Cell Lung Cancer**	
A549/ATCC	102.19
EKVX	100.47
HOP-62	106.54
HOP-92	97.51
NCI-H226	100.91
NCI-H23	100.96
NCI-H322M	97.15
NCI-H460	100.17
NCI-H522	89.73
**Colon Cancer**	
COLO 205	117.56
HCC-2998	102.53
HCT-116	103.06
HCT-15	99.16
HT29	110.16
KM12	99.03
SW-620	104.70
**CNS Cancer**	
SF-268	98.97
SF-295	103.35
SF-539	95.16
SNB-19	99.84
SNB-75	95.83
U251	97.38
**Melanoma**	
LOX IMVI	98.00
MALME-3M	95.69
M14	105.52
MDA-MB-435	98.19
SK-MEL-2	95.28
SK-MEL-28	102.35
SK-MEL-5	98.02
UACC-257	99.67
UACC-62	98.74
**Ovarian Cancer**	
IGROV1	100.67
OVCAR-3	110.48
OVCAR-4	100.76
OVCAR-5	107.22
OVCAR-8	103.61
NCI/ADR-RES	108.46
SK-OV-3	101.04
**Renal Cancer**	
786-0	105.86
A498	107.20
ACHN	101.32
CAKI-1	101.22
RXF 393	110.86
SN12C	99.69
TK-10	110.23
UO-31	94.17
**Prostate Cancer**	
DU-145	104.52
**Breast Cancer**	
MCF7	93.10
MDA-MB-231/ATCC	106.28
HS 578T	96.11
BT-549	110.36
T-47D	93.90
MDA-MB-468	109.33
Mean	101.51
Delta	11.78
Range	27.83

Mean Growth Percent - Growth Percent

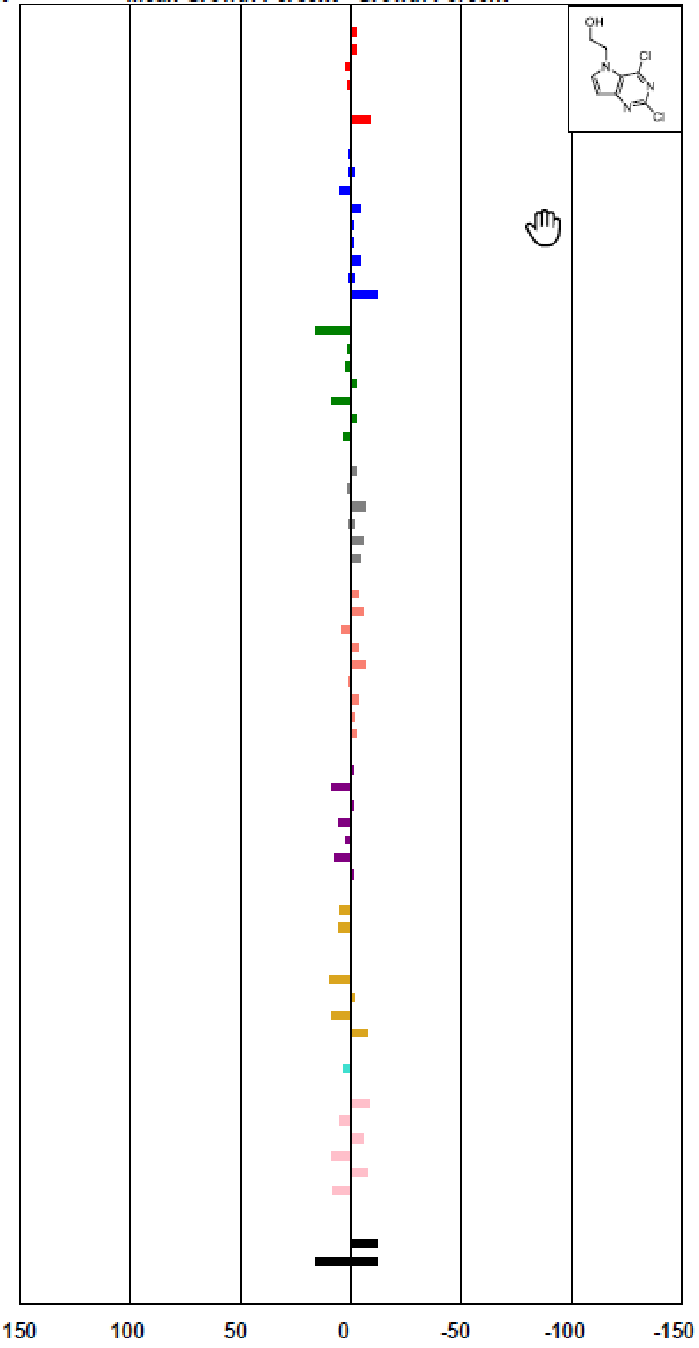

Figure S2. ^{1}H NMR Spectra

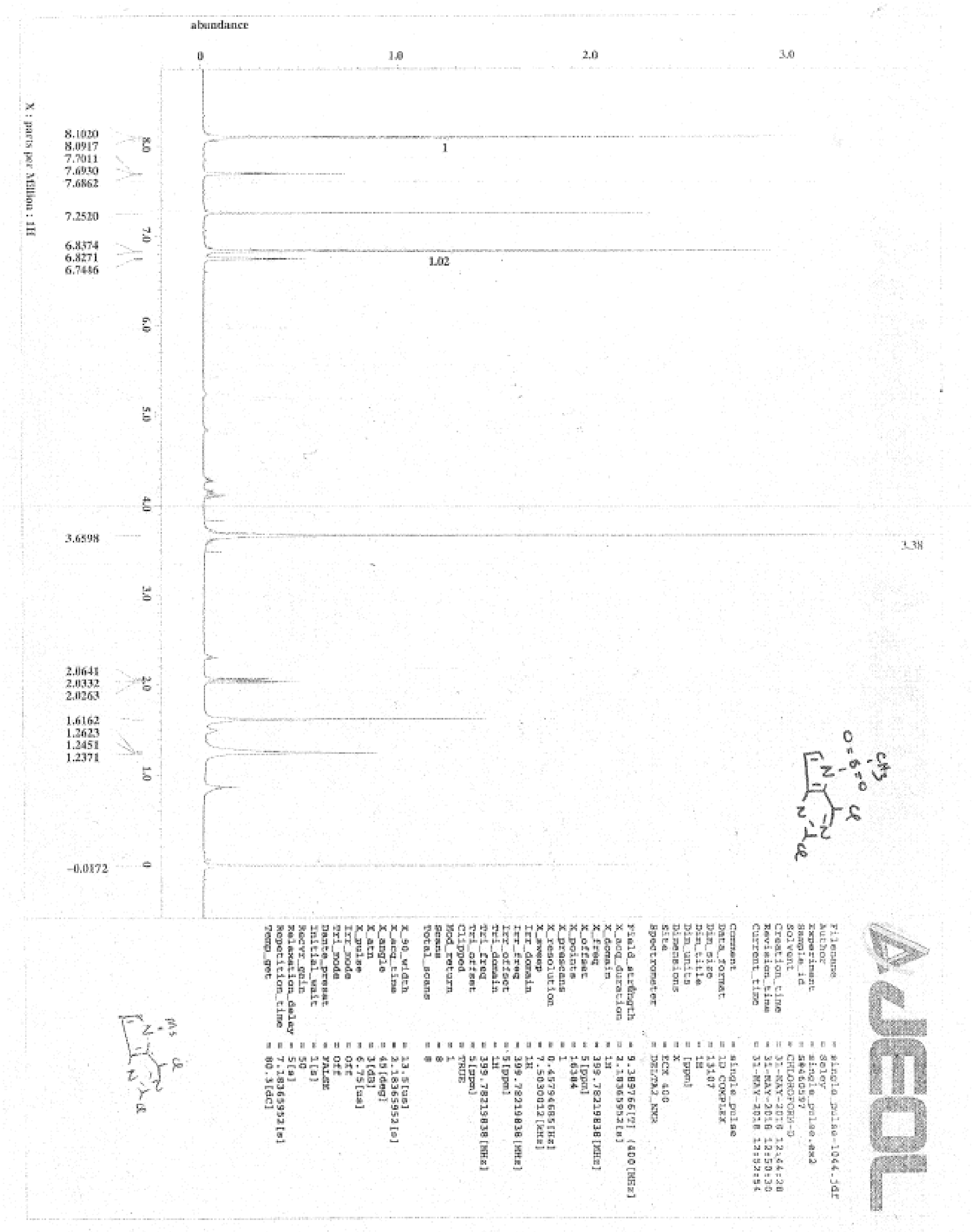

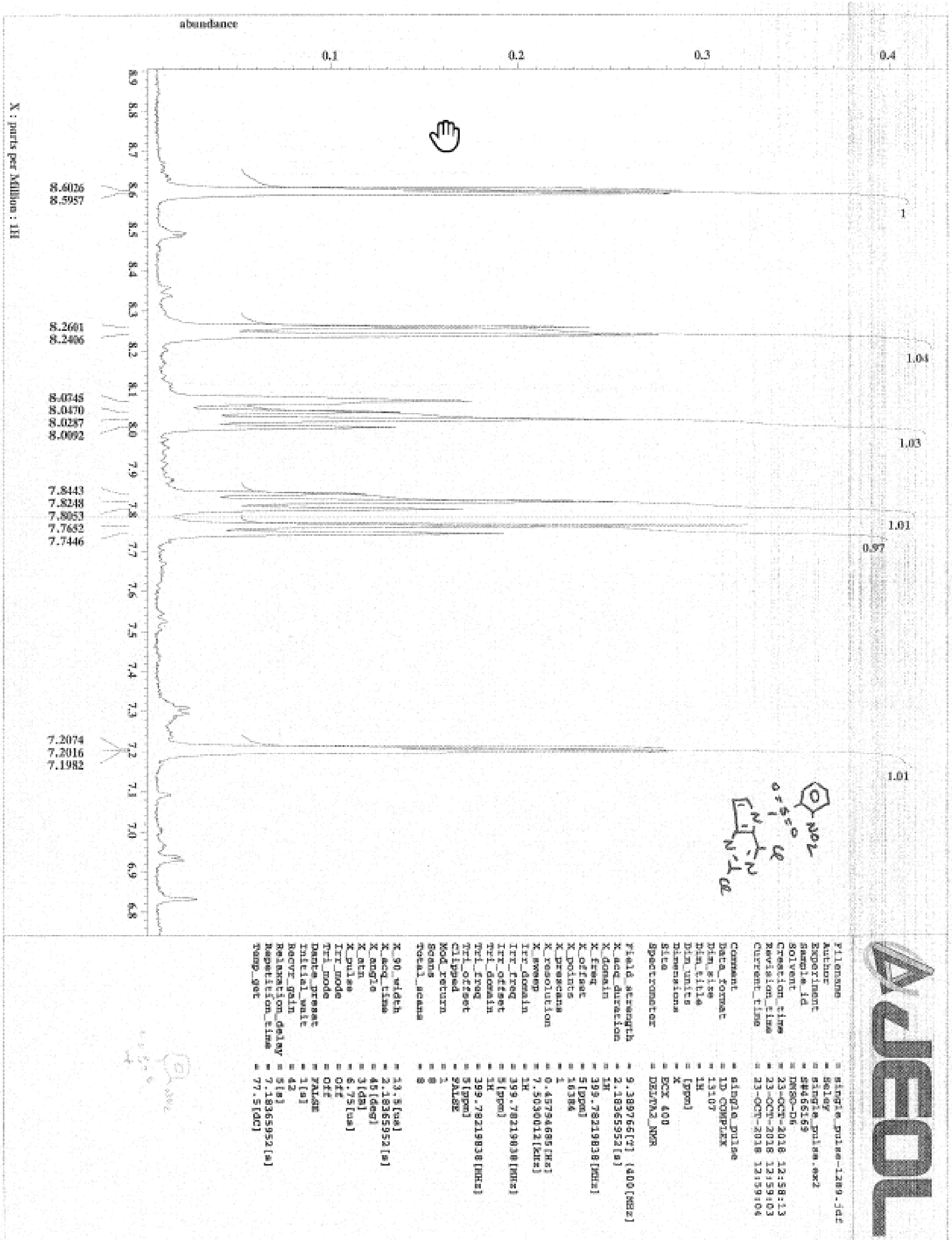
abundance
0.1
0.2
0.3
0.4
X : parts per Million : 1H
8.6026
8.5957
8.2601
8.2406
8.0745
8.0470
8.0287
8.0092
7.8443
7.8248
7.8053
7.7652
7.7446
7.2074
7.2016
7.1982
1
1.04
1.03
1.01
0.97
1.01
JEOL
Filename = single_pulse-1289.jdf
Author = Seley
Experiment = single_pulse.ex2
Sample_id = S#466169
Solvent = DMSO-D6
Creation_time = 23-OCT-2018 12:58:13
Revision_time = 23-OCT-2018 12:59:03
Current_time = 23-OCT-2018 12:59:04
Comment = single_pulse
Data_format = 1D COMPLEX
Dim_size = 13107
Dim_title = 1H
Dim_units = [ppm]
Dimensions = X
Site = ECX 400
Spectrometer = DELTA2_NMR
Field_strength = 9.389766[T] (400[MHz]
X_acq_duration = 2.18365952[s]
X_domain = 1H
X_freq = 399.78219838[MHz]
X_offset = 5[ppm]
X_points = 16384
X_prescans = 1
X_resolution = 0.45794685[Hz]
X_sweep = 7.5030012[kHz]
Irr_domain = 1H
Irr_freq = 399.78219838[MHz]
Irr_offset = 5[ppm]
Tri_domain = 1H
Tri_freq = 399.78219838[MHz]
Tri_offset = 5[ppm]
Clipped = FALSE
Mod_return = 1
Scans = 8
Total_scans = 8
X_90_width = 13.5[us]
X_acq_time = 2.18365952[s]
X_angle = 45[deg]
X_atn = 3[dB]
X_pulse = 6.75[us]
Irr_mode = Off
Tri_mode = Off
Dante_presat = FALSE
Initial_wait = 1[s]
Recvr_gain = 42
Relaxation_delay = 5[s]
Repetition_time = 7.18365952[s]
Temp_get = 77.5[dC]

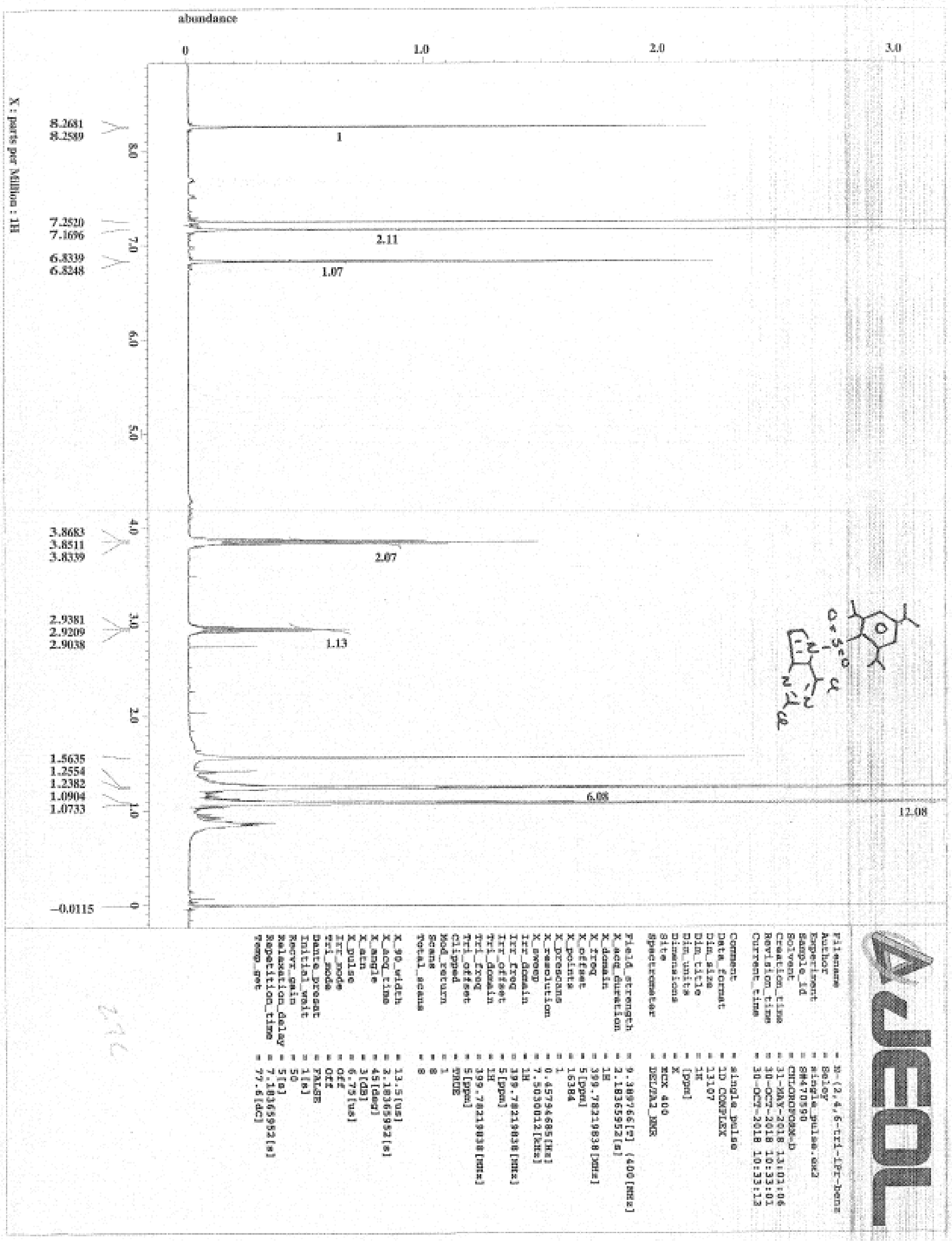

abundance
0
1.0
2.0
3.0
X : parts per Million : 1H
8.2681
8.2589
7.2520
7.1696
6.8339
6.8248
3.8683
3.8511
3.8339
2.9381
2.9209
2.9038
1.5635
1.2554
1.2382
1.0904
1.0733
-0.0115
8.0
7.0
6.0
5.0
4.0
3.0
2.0
1.0
0
1
2.11
1.07
2.07
1.13
6.08
12.08
JEOL
Filename = N-(2,4,6-tri-iPr-bens
Author = Seley
Experiment = single_pulse.ex2
Sample_id = SM470590
Solvent = CHLOROFORM-D
Creation_time = 31-MAY-2018 13:01:06
Revision_time = 30-OCT-2018 10:33:01
Current_time = 30-OCT-2018 10:33:13
Comment = single_pulse
Data_format = 1D COMPLEX
Dim_size = 13107
Dim_title = 1H
Dim_units = [ppm]
Dimensions = X
Site = ECX 400
Spectrometer = DELTA2_NMR
Field_strength = 9.389766[T] (400[MHz])
X_acq_duration = 2.18365952[s]
X_domain = 1H
X_freq = 399.78219838[MHz]
X_offset = 5[ppm]
X_points = 16384
X_prescans = 1
X_resolution = 0.45794685[Hz]
X_sweep = 7.50300012[kHz]
Irr_domain = 1H
Irr_freq = 399.78219838[MHz]
Irr_offset = 5[ppm]
Tri_domain = 1H
Tri_freq = 399.78219838[MHz]
Tri_offset = 5[ppm]
Clipped = TRUE
Mod_return = 1
Scans = 8
Total_scans = 8
X_90_width = 13.5[us]
X_acq_time = 2.18365952[s]
X_angle = 45[deg]
X_atn = 3[dB]
X_pulse = 6.75[us]
Irr_mode = Off
Tri_mode = Off
Dante_presat = FALSE
Initial_wait = 1[s]
Recvr_gain = 50
Relaxation_delay = 5[s]
Repetition_time = 7.18365952[s]
Temp_get = 77.6[dC]

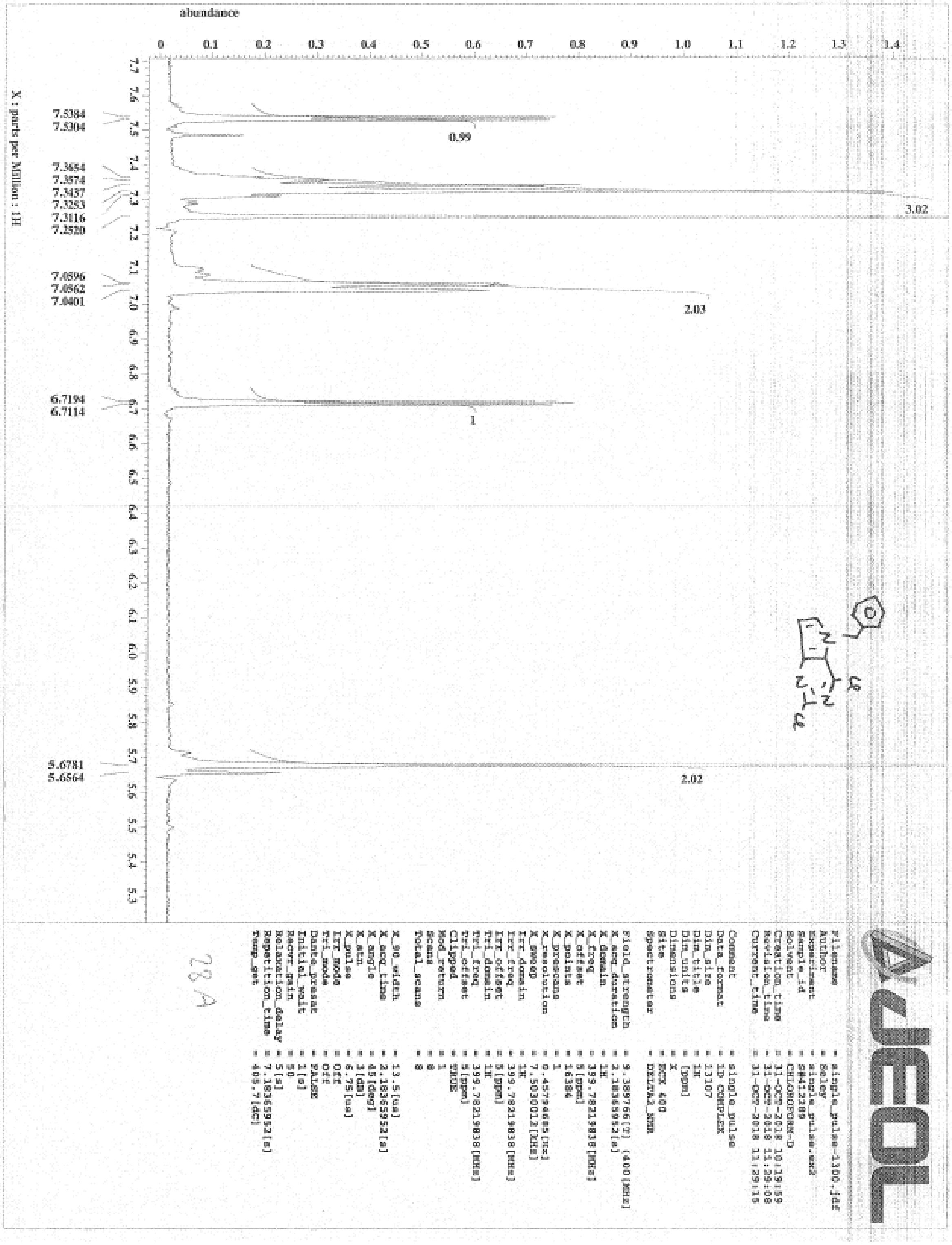

abundance
X : parts per Million : 1H
7.5384
7.5304
7.3654
7.3574
7.3437
7.3253
7.3116
7.2520
7.0596
7.0562
7.0401
6.7194
6.7114
5.6781
5.6564
0.99
3.02
2.03
1
2.02
JEOL
28A

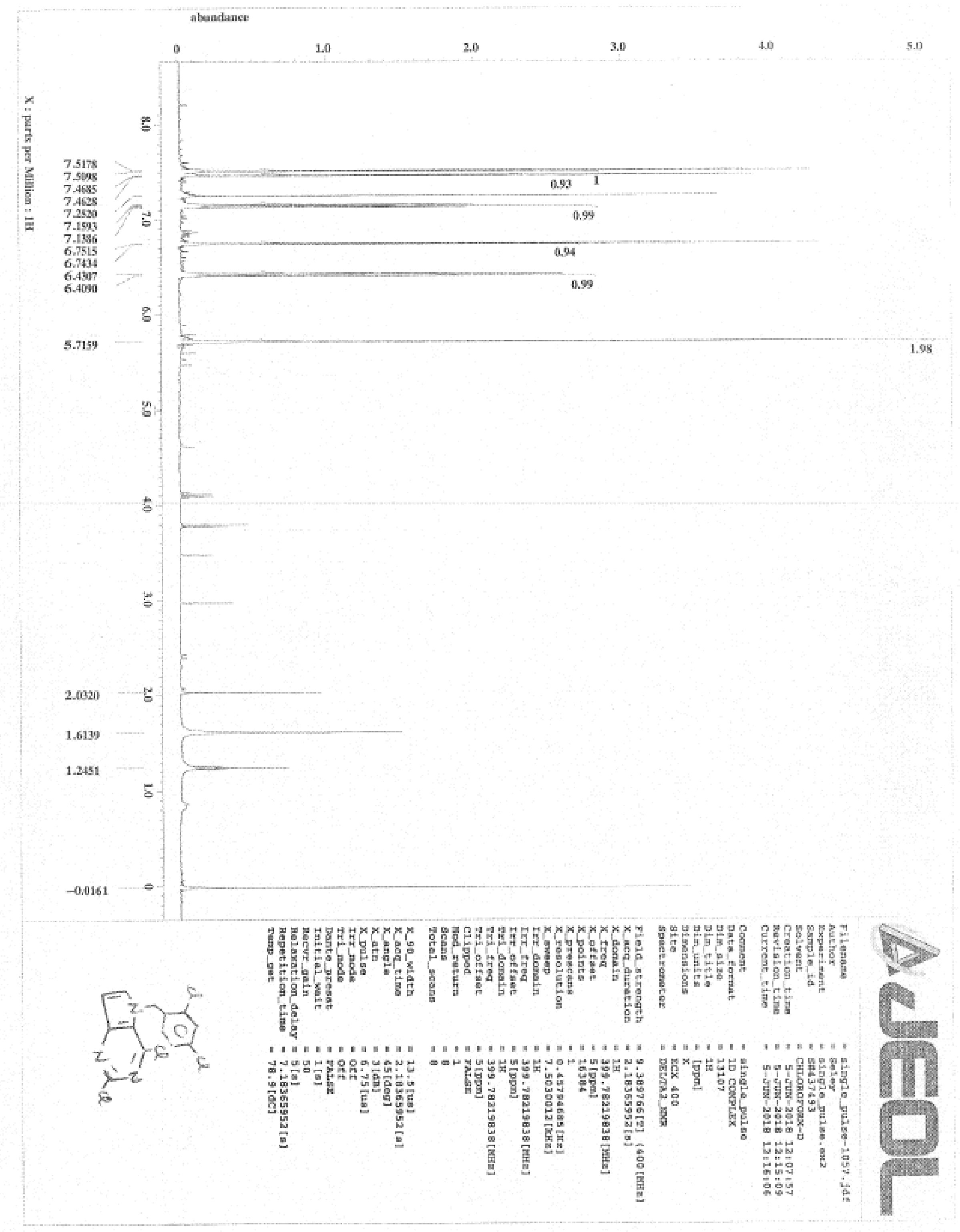

abundance
X : parts per Million : 1H
7.5178
7.5098
7.4685
7.4628
7.2520
7.1593
7.1386
6.7515
6.7434
6.4307
6.4090
5.7159
2.0320
1.6139
1.2451
-0.0161
0.93
1
0.99
0.94
0.99
1.98
JEOL

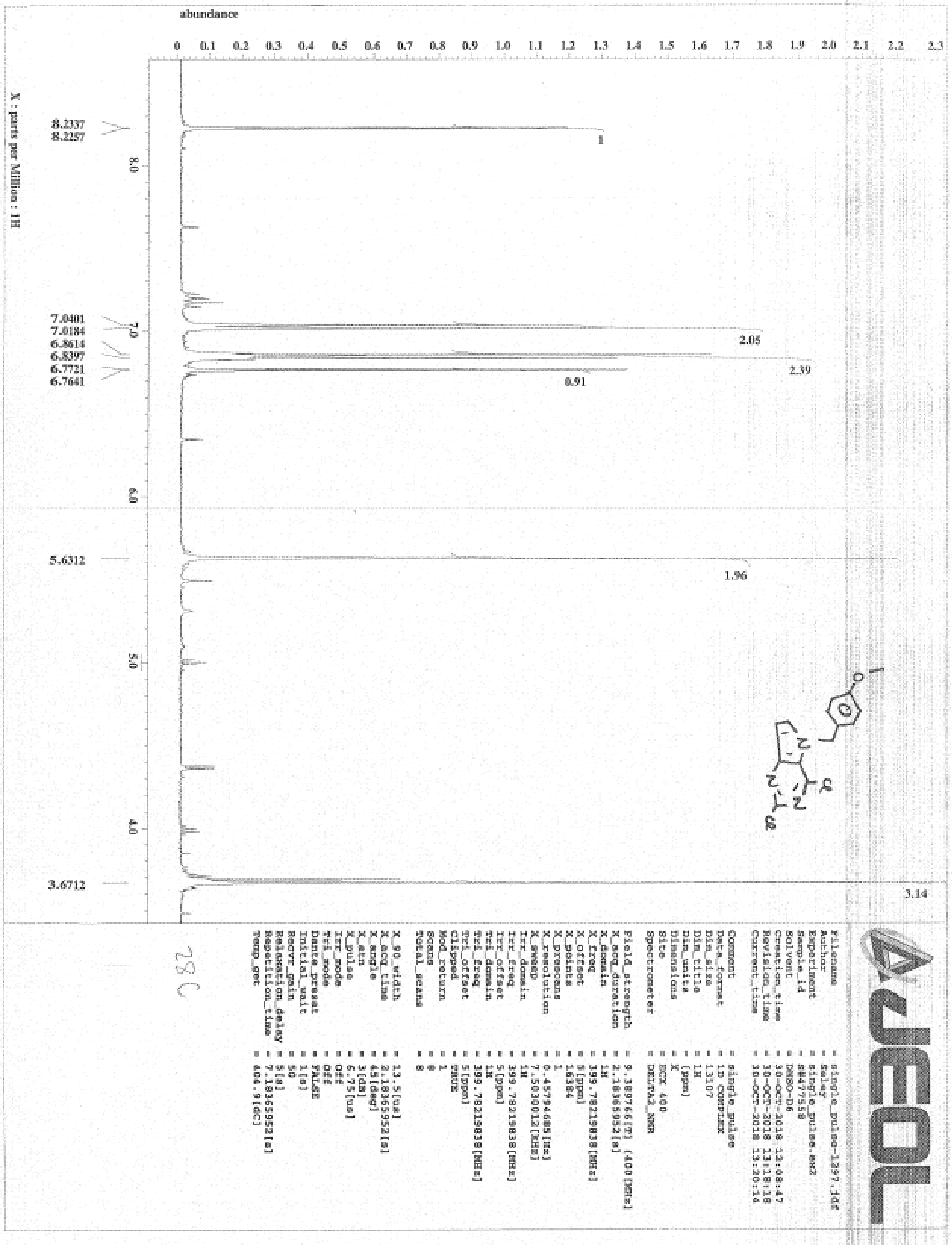
abundance
X : parts per Million : 1H
8.2337
8.2257
7.0401
7.0184
6.8614
6.8397
6.7721
6.7641
5.6312
3.6712
1
2.05
2.39
0.91
1.96
3.14

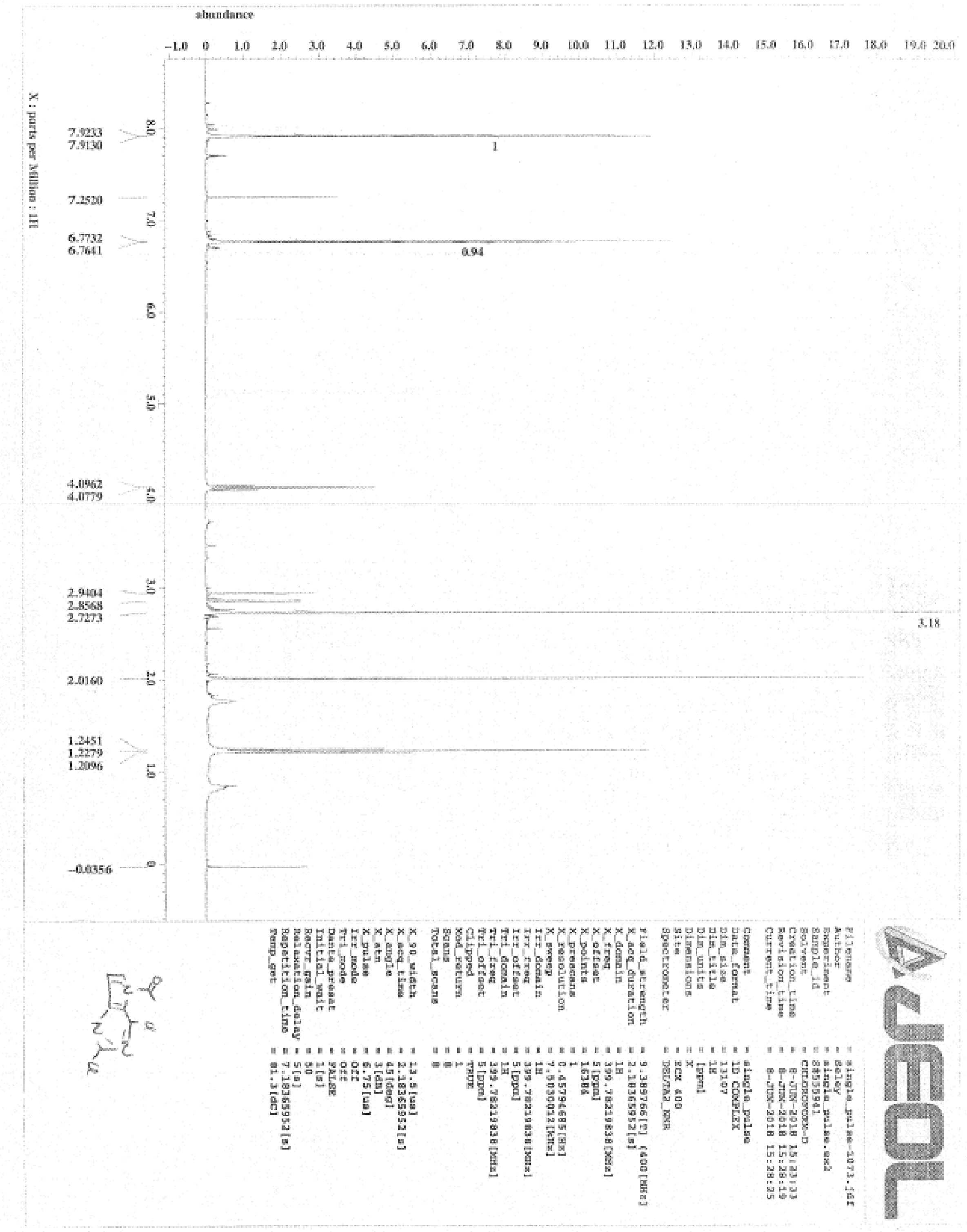
abundance
X : parts per Million : 1H
7.9233
7.9130
7.2520
6.7732
6.7641
4.0962
4.0779
2.9404
2.8568
2.7273
2.0160
1.2451
1.2279
1.2096
-0.0356
1
0.94
3.18

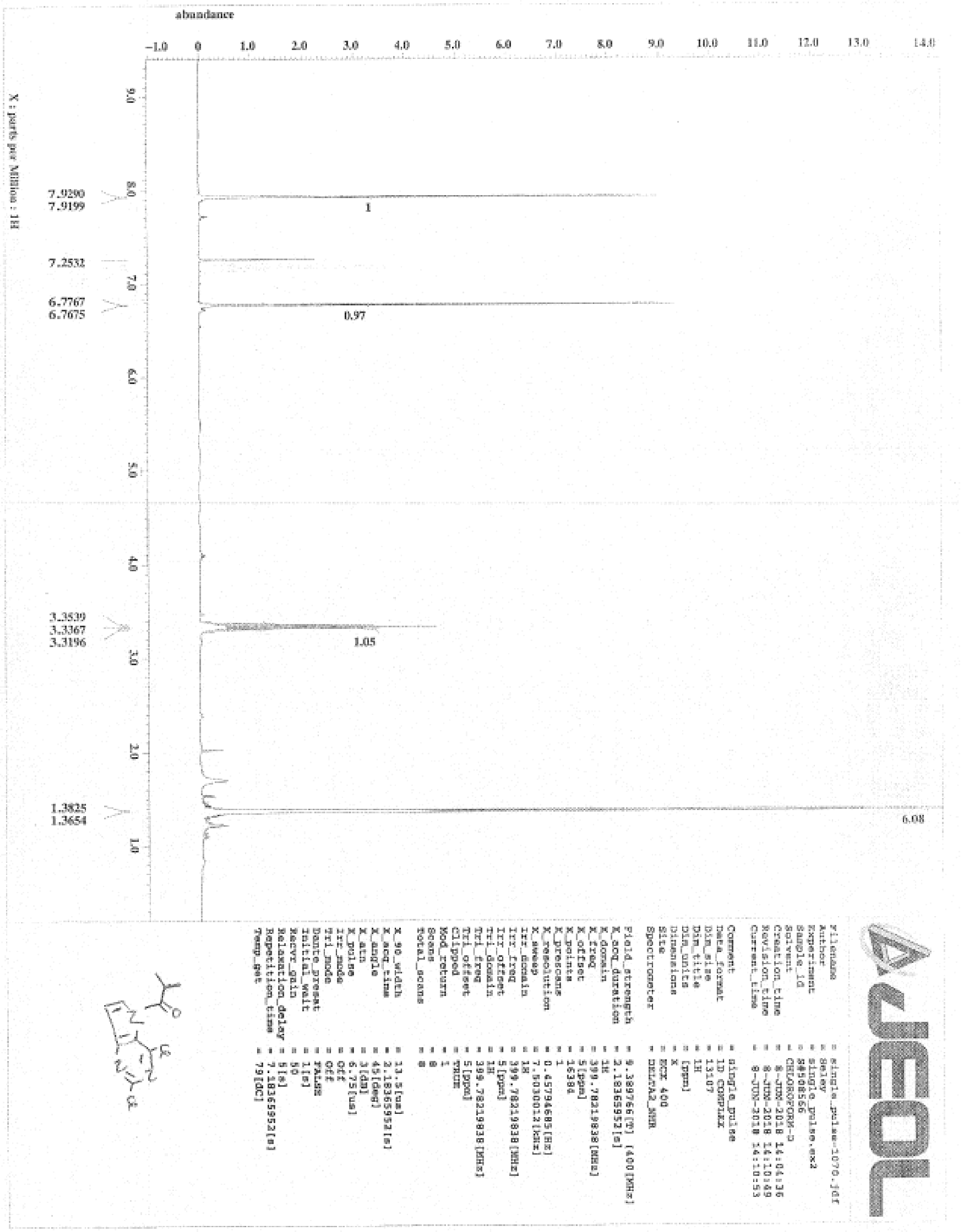
abundance
X : parts per Million : 1H
7.9290
7.9199
7.2532
6.7767
6.7675
3.3539
3.3367
3.3196
1.3825
1.3654
1
0.97
1.05
6.08
JEOL

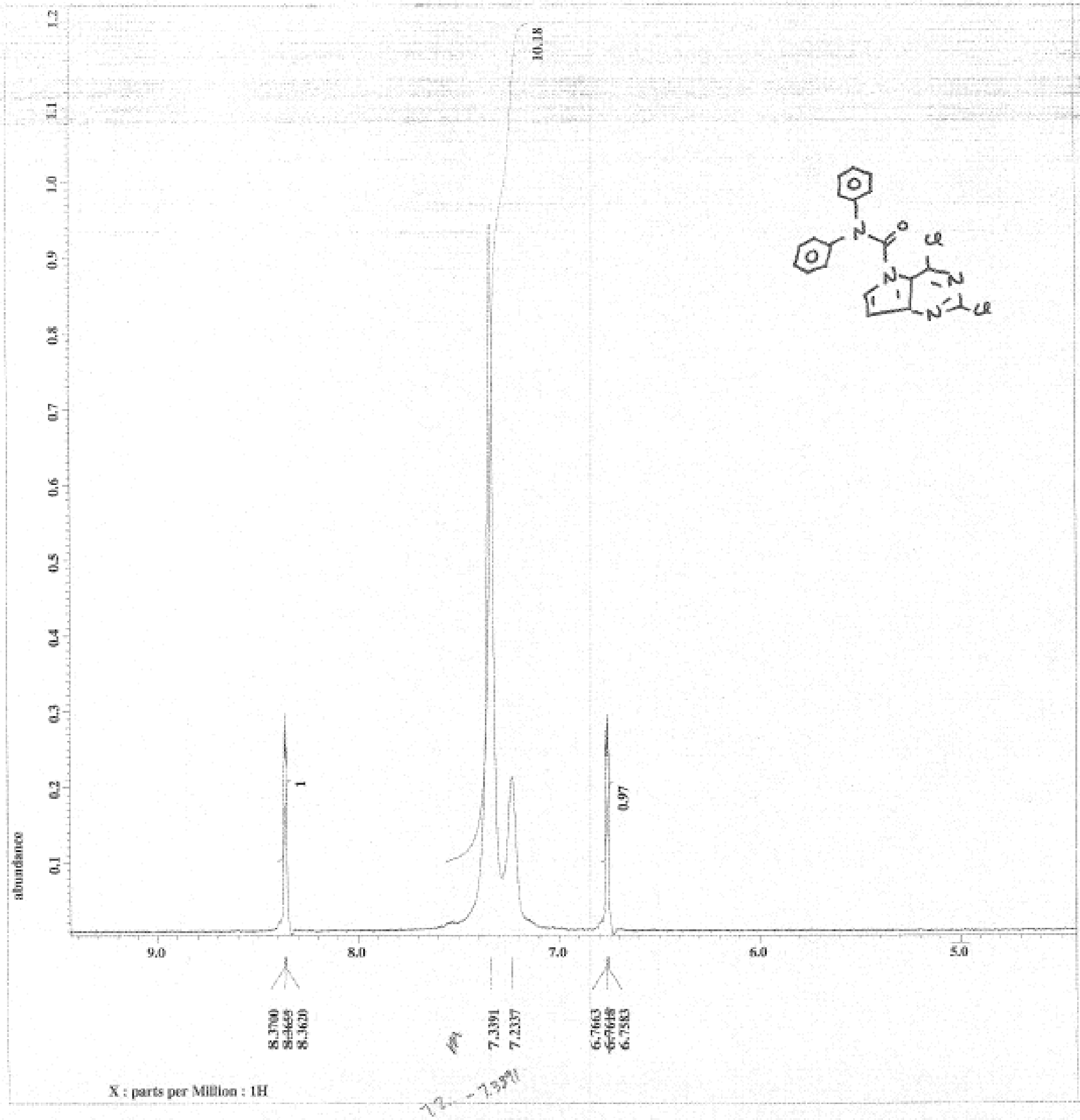


```
Filename          = single_pulse-1284.jdf
Author            = Seley
Experiment        = single_pulse.ex2
Sample_id         = 8#540250
Solvent           = DMSO-D6
Creation_time     = 16-OCT-2018 15:03:15
Revision_time     = 16-OCT-2018 15:07:54
Current_time      = 16-OCT-2018 15:07:55

Comment           = single_pulse
Data_format       = 1D COMPLEX
Dim_size          = 13107
Dim_title         = 1H
Dim_units         = [ppm]
Dimensions        = X
Site              = ECX 400
Spectrometer      = DELTA2_NMR

Field_strength    = 9.389766[T] (400[MHz]
X_acq_duration    = 2.18365952[s]
X_domain          = 1H
X_freq            = 399.78219838[MHz]
X_offset          = 5[ppm]
X_points          = 16384
X_prescans        = 1
X_resolution      = 0.45794685[Hz]
X_sweep           = 7.5030012[kHz]
Irr_domain        = 1H
Irr_freq          = 399.78219838[MHz]
Irr_offset        = 5[ppm]
Tri_domain        = 1H
Tri_freq          = 399.78219838[MHz]
Tri_offset        = 5[ppm]
Clipped           = FALSE
Mod_return        = 1
Scans             = 8
Total_scans       = 8

X_90_width        = 13.5[us]
X_acq_time        = 2.18365952[s]
X_angle           = 45[deg]
X_atn             = 3[dB]
X_pulse           = 6.75[us]
Irr_mode          = Off
Tri_mode          = Off
Dante_presat      = FALSE
Initial_wait      = 1[s]
Recvr_gain        = 46
Relaxation_delay  = 5[s]
Repetition_time   = 7.18365952[s]
Temp_get          = 77.1[dC]
```

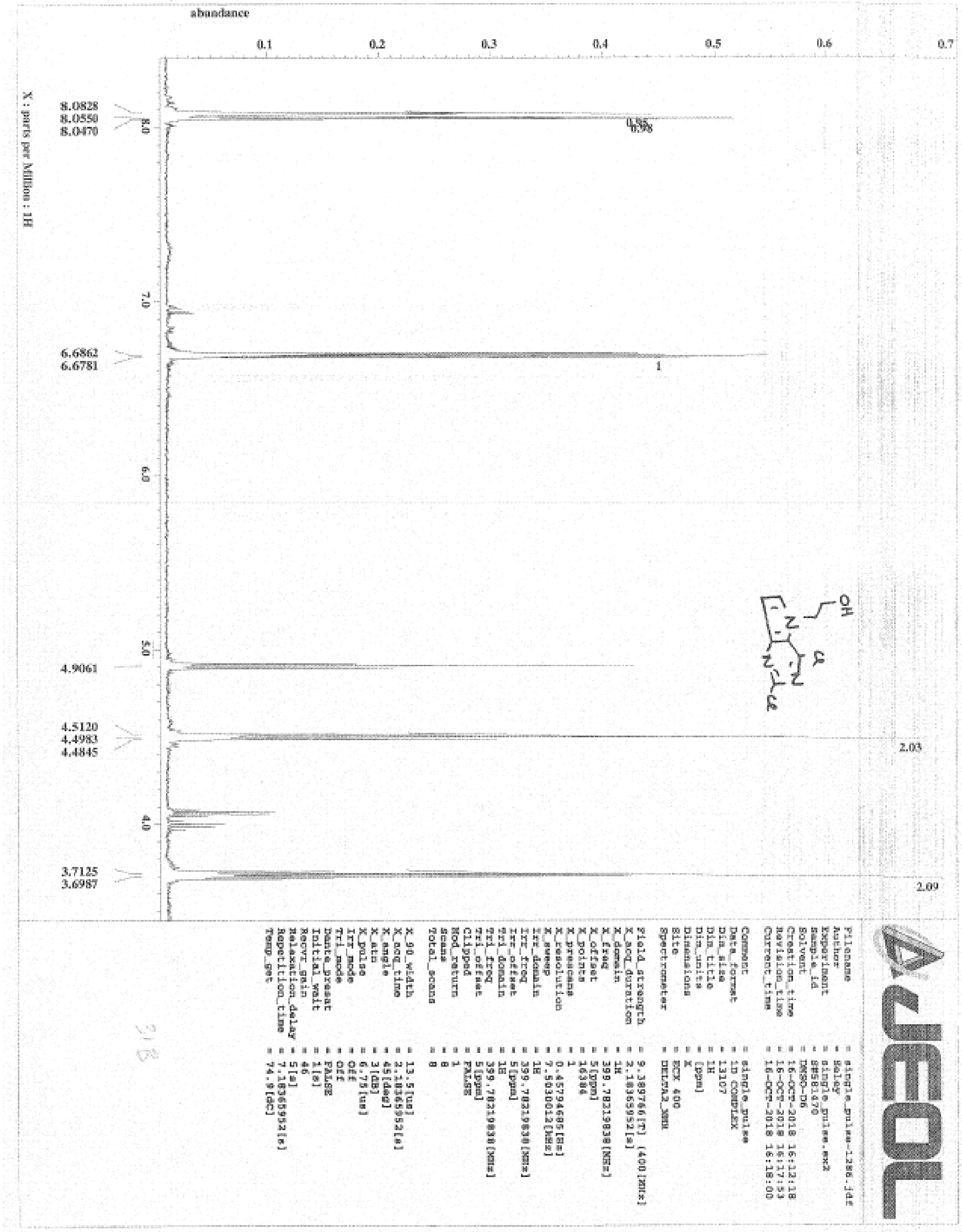
abundance
X : parts per Million : 1H
8.0828
8.0550
8.0470
6.6862
6.6781
4.9061
4.5120
4.4983
4.4845
3.7125
3.6987
0.95
0.98
1
2.03
2.09
JEOL

Figure S3. ^{13}C NMR Spectra

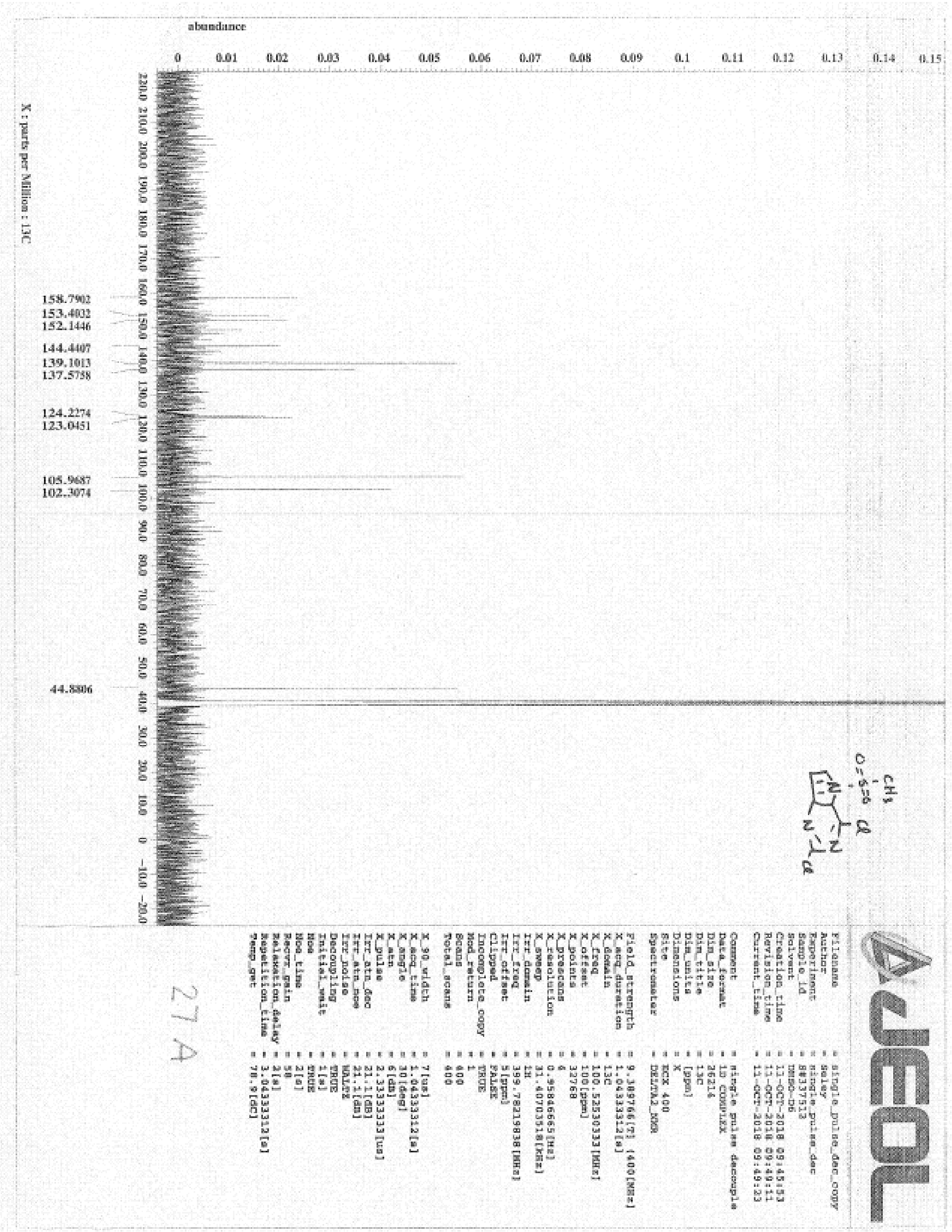

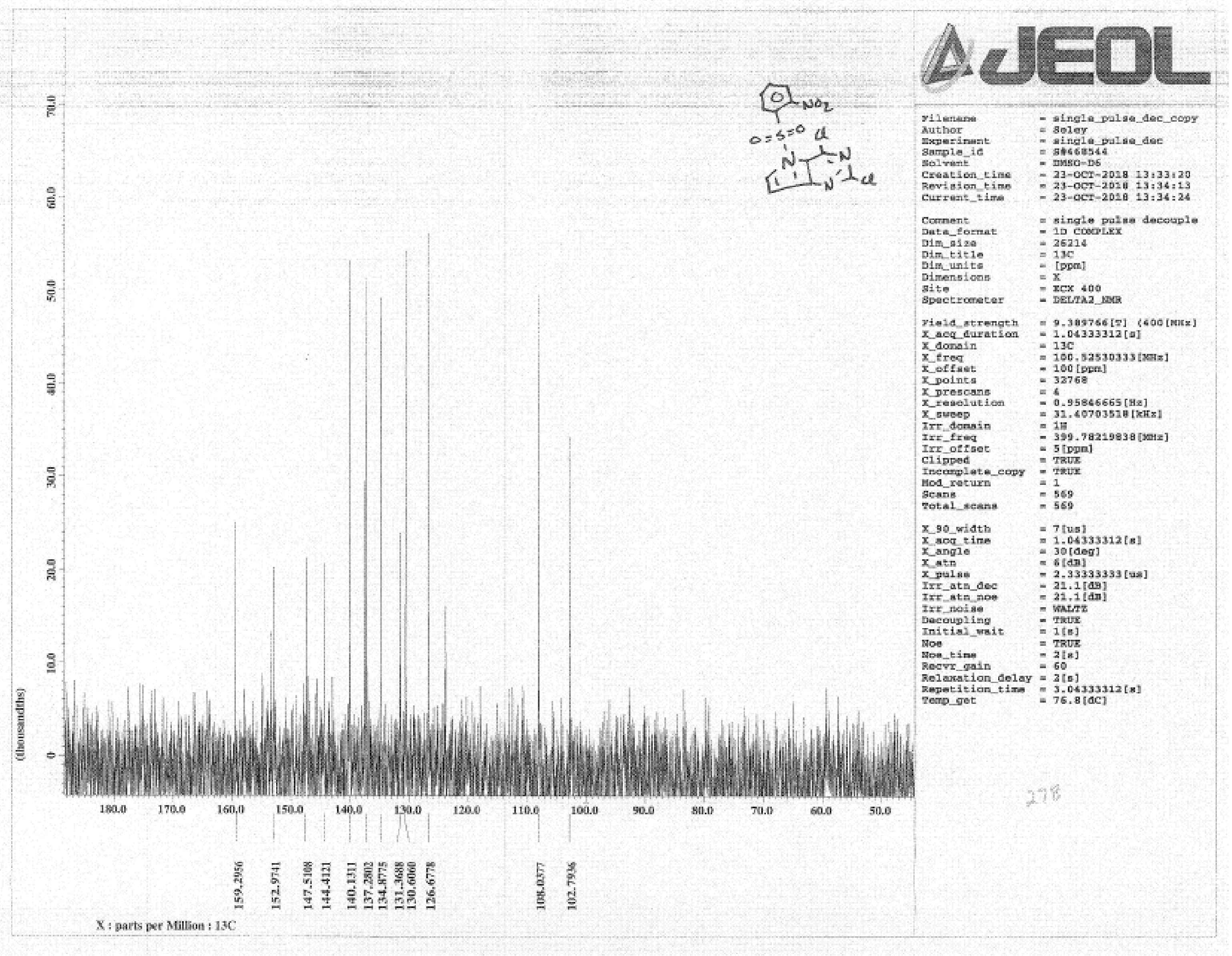
(thousandths)
X : parts per Million : 13C
159.2956
152.9741
147.5108
144.4121
140.1311
137.2802
134.8775
131.3688
130.6060
126.6778
108.0377
102.7936
JEOL
Filename = single_pulse_dec_copy
Author = Soley
Experiment = single_pulse_dec
Sample_id = S#468544
Solvent = DMSO-D6
Creation_time = 23-OCT-2018 13:33:20
Revision_time = 23-OCT-2018 13:34:13
Current_time = 23-OCT-2018 13:34:24
Comment = single pulse decouple
Data_format = 1D COMPLEX
Dim_size = 26214
Dim_title = 13C
Dim_units = [ppm]
Dimensions = X
Site = ECX 400
Spectrometer = DELTA2_NMR
Field_strength = 9.389766[T] (400[MHz])
X_acq_duration = 1.04333312[s]
X_domain = 13C
X_freq = 100.52530333[MHz]
X_offset = 100[ppm]
X_points = 32768
X_prescans = 4
X_resolution = 0.95846665[Hz]
X_sweep = 31.40703518[kHz]
Irr_domain = 1H
Irr_freq = 399.78219838[MHz]
Irr_offset = 5[ppm]
Clipped = TRUE
Incomplete_copy = TRUE
Mod_return = 1
Scans = 569
Total_scans = 569
X_90_width = 7[us]
X_acq_time = 1.04333312[s]
X_angle = 30[deg]
X_atn = 6[dB]
X_pulse = 2.33333333[us]
Irr_atn_dec = 21.1[dB]
Irr_atn_noe = 21.1[dB]
Irr_noise = WALTZ
Decoupling = TRUE
Initial_wait = 1[s]
Noe = TRUE
Noe_time = 2[s]
Recvr_gain = 60
Relaxation_delay = 2[s]
Repetition_time = 3.04333312[s]
Temp_get = 76.8[dC]
278

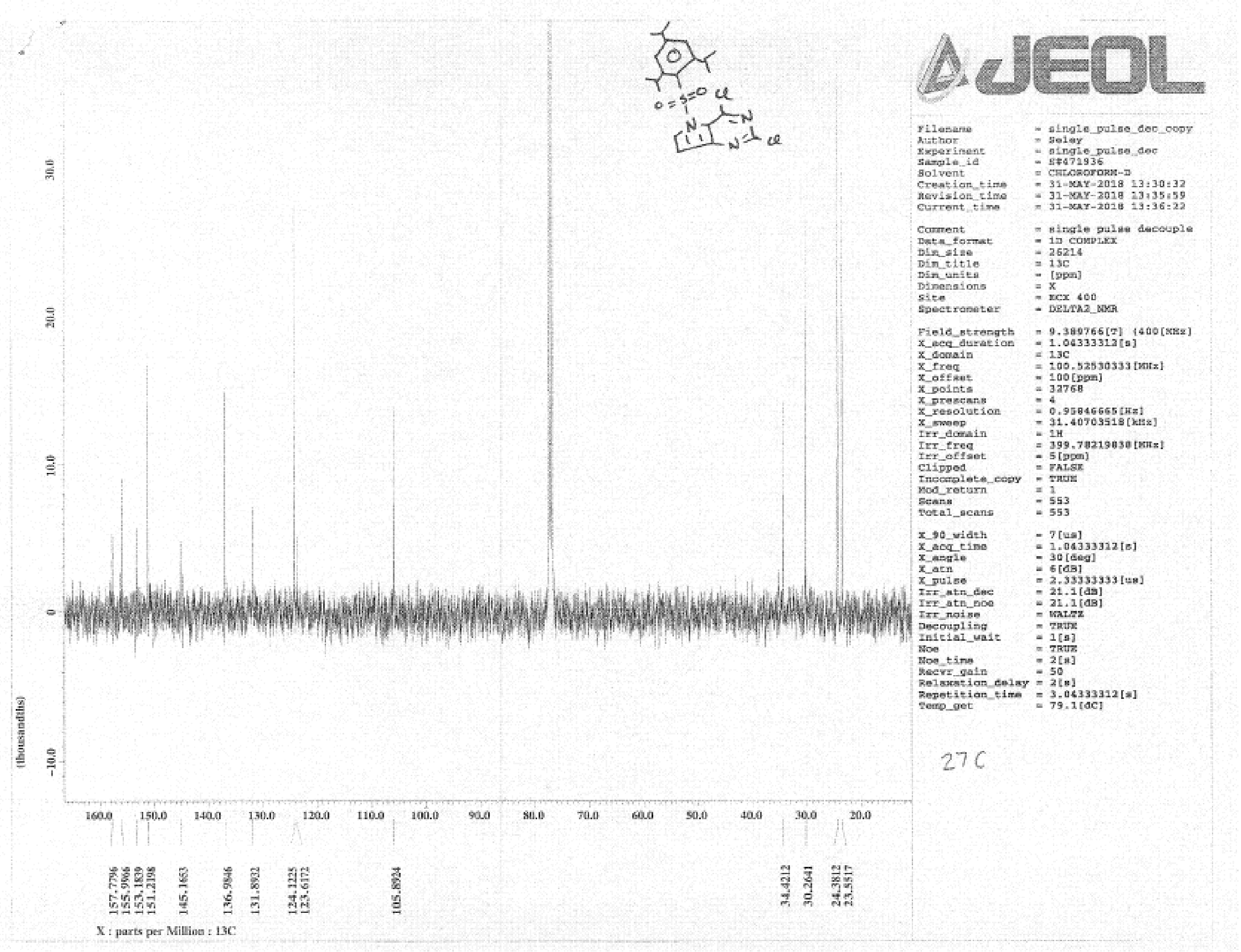
JEOL
(thousandths)
-10.0
0
10.0
20.0
30.0
160.0
150.0
140.0
130.0
120.0
110.0
100.0
90.0
80.0
70.0
60.0
50.0
40.0
30.0
20.0
157.7796
155.9966
153.1839
151.2198
145.1653
136.9846
131.8932
124.1225
123.6172
105.8924
34.4212
30.2641
24.3812
23.5517
X : parts per Million : 13C
Filename = single_pulse_dec_copy
Author = Seley
Experiment = single_pulse_dec
Sample_id = S#471936
Solvent = CHLOROFORM-D
Creation_time = 31-MAY-2018 13:30:32
Revision_time = 31-MAY-2018 13:35:59
Current_time = 31-MAY-2018 13:36:22
Comment = single pulse decouple
Data_format = 1D COMPLEX
Dim_size = 26214
Dim_title = 13C
Dim_units = [ppm]
Dimensions = X
Site = ECX 400
Spectrometer = DELTA2_NMR
Field_strength = 9.389766[T] (400[MHz]
X_acq_duration = 1.04333312[s]
X_domain = 13C
X_freq = 100.52530333[MHz]
X_offset = 100[ppm]
X_points = 32768
X_prescans = 4
X_resolution = 0.95846665[Hz]
X_sweep = 31.40703518[kHz]
Irr_domain = 1H
Irr_freq = 399.78219830[MHz]
Irr_offset = 5[ppm]
Clipped = FALSE
Incomplete_copy = TRUE
Mod_return = 1
Scans = 553
Total_scans = 553
X_90_width = 7[us]
X_acq_time = 1.04333312[s]
X_angle = 30[deg]
X_atn = 6[dB]
X_pulse = 2.33333333[us]
Irr_atn_dec = 21.1[dB]
Irr_atn_noe = 21.1[dB]
Irr_noise = WALTZ
Decoupling = TRUE
Initial_wait = 1[s]
Noe = TRUE
Noe_time = 2[s]
Recvr_gain = 50
Relaxation_delay = 2[s]
Repetition_time = 3.04333312[s]
Temp_get = 79.1[dC]
27C

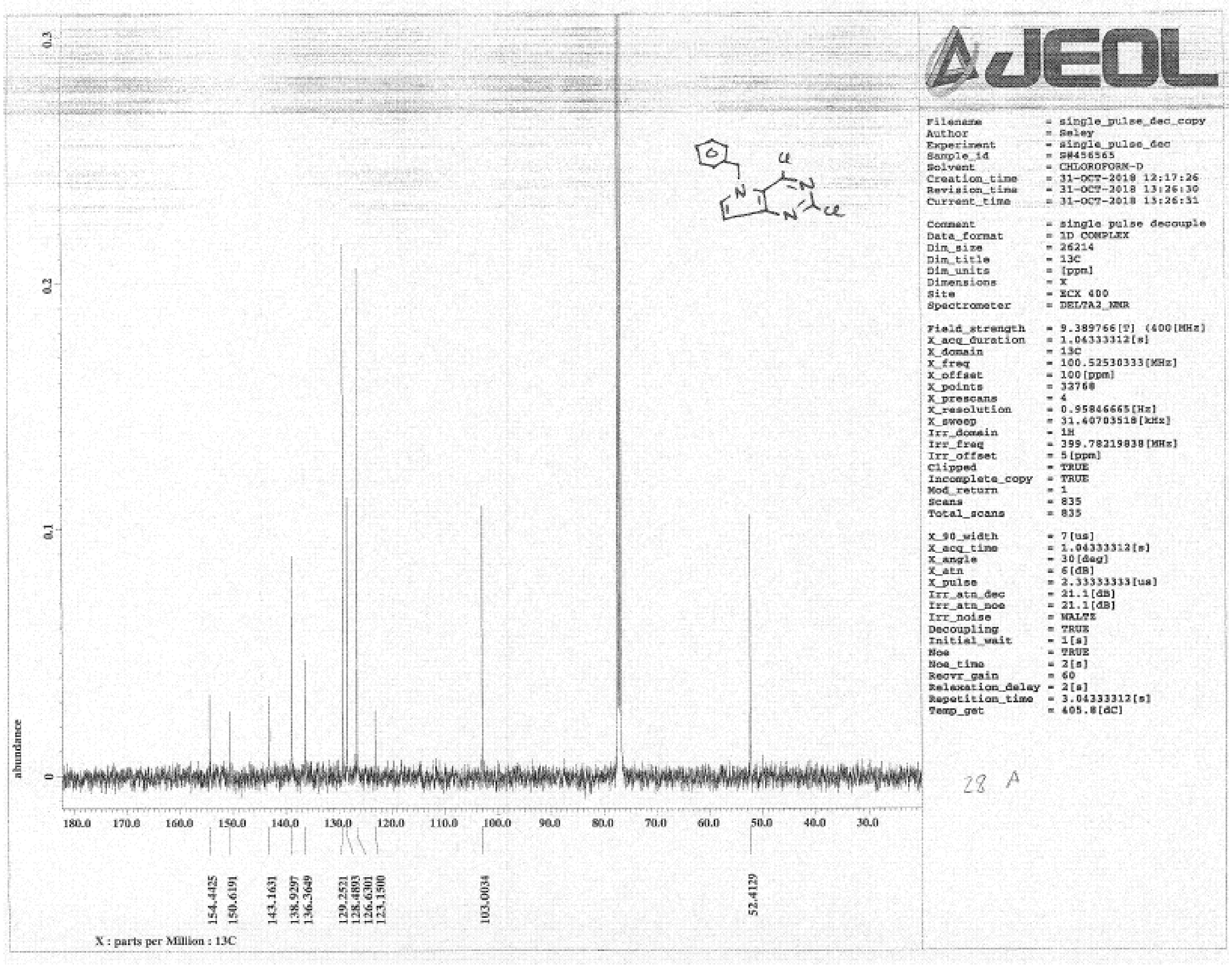

JEOL
abundance
0
0.1
0.2
0.3
180.0 170.0 160.0 150.0 140.0 130.0 120.0 110.0 100.0 90.0 80.0 70.0 60.0 50.0 40.0 30.0
X : parts per Million : 13C
154.4425
150.6191
143.1631
138.9297
136.3649
129.2521
128.4893
126.6301
123.1500
103.0034
52.4129
Filename = single_pulse_dec_copy
Author = Seley
Experiment = single_pulse_dec
Sample_id = SW456565
Solvent = CHLOROFORM-D
Creation_time = 31-OCT-2018 12:17:26
Revision_time = 31-OCT-2018 13:26:30
Current_time = 31-OCT-2018 13:26:31
Comment = single pulse decouple
Data_format = 1D COMPLEX
Dim_size = 26214
Dim_title = 13C
Dim_units = [ppm]
Dimensions = X
Site = ECX 400
Spectrometer = DELTA2_NMR
Field_strength = 9.389766[T] (400[MHz]
X_acq_duration = 1.04333312[s]
X_domain = 13C
X_freq = 100.52530333[MHz]
X_offset = 100[ppm]
X_points = 32768
X_prescans = 4
X_resolution = 0.95846665[Hz]
X_sweep = 31.40703518[kHz]
Irr_domain = 1H
Irr_freq = 399.78219838[MHz]
Irr_offset = 5[ppm]
Clipped = TRUE
Incomplete_copy = TRUE
Mod_return = 1
Scans = 835
Total_scans = 835
X_90_width = 7[us]
X_acq_time = 1.04333312[s]
X_angle = 30[deg]
X_atn = 6[dB]
X_pulse = 2.33333333[us]
Irr_atn_dec = 21.1[dB]
Irr_atn_noe = 21.1[dB]
Irr_noise = WALTZ
Decoupling = TRUE
Initial_wait = 1[s]
Noe = TRUE
Noe_time = 2[s]
Recvr_gain = 60
Relaxation_delay = 2[s]
Repetition_time = 3.04333312[s]
Temp_get = 405.8[dC]
28 A

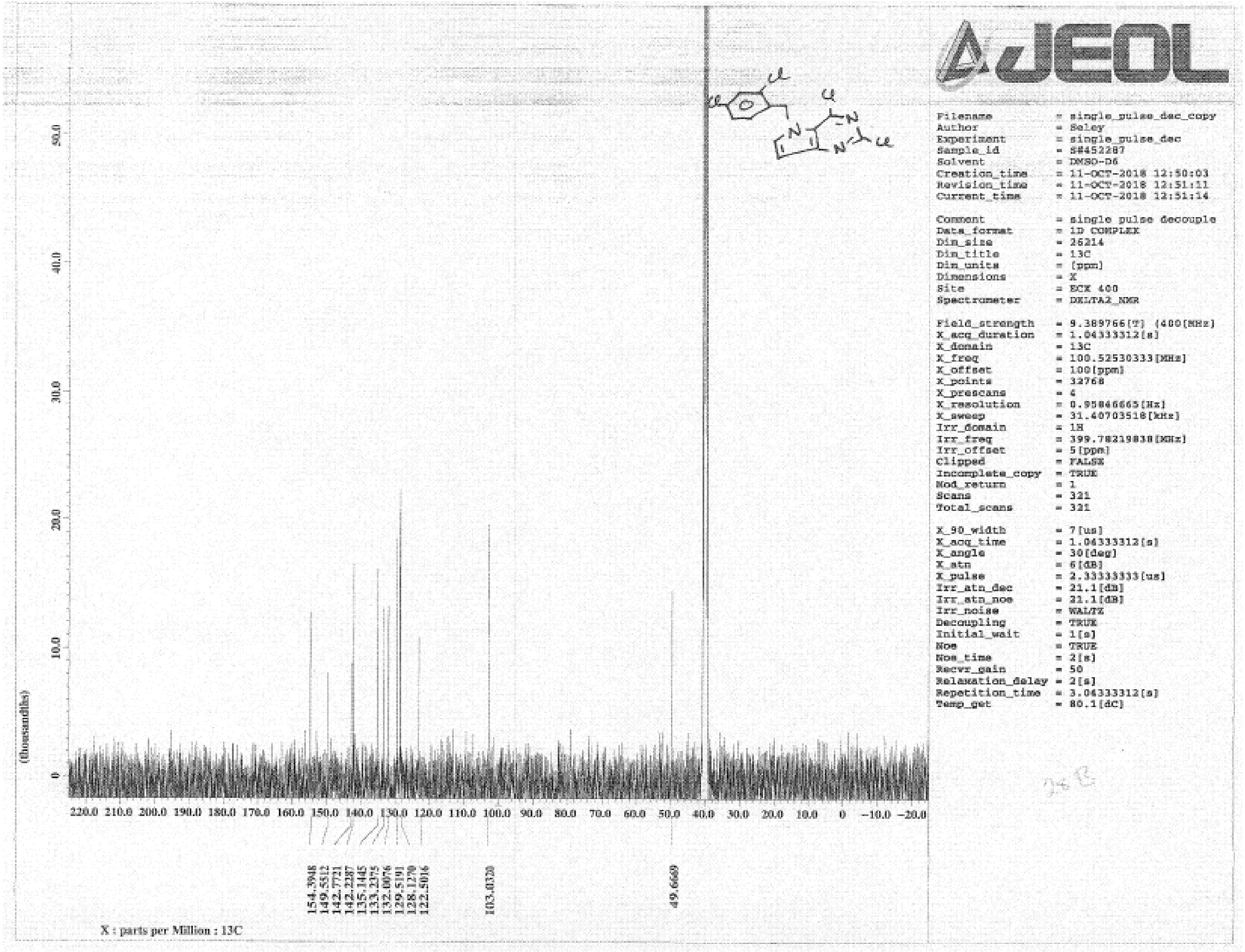

(thousandths)
50.0
40.0
30.0
20.0
10.0
0
220.0 210.0 200.0 190.0 180.0 170.0 160.0 150.0 140.0 130.0 120.0 110.0 100.0 90.0 80.0 70.0 60.0 50.0 40.0 30.0 20.0 10.0 0 −10.0 −20.0
154.3948
149.5512
142.7721
142.2287
135.1445
133.2375
132.0076
129.5191
128.1270
122.5016
103.0320
49.6669
X : parts per Million : 13C
JEOL
Filename = single_pulse_dec_copy
Author = Soley
Experiment = single_pulse_dec
Sample_id = S#452287
Solvent = DMSO-D6
Creation_time = 11-OCT-2018 12:50:03
Revision_time = 11-OCT-2018 12:51:11
Current_time = 11-OCT-2018 12:51:14
Comment = single pulse decouple
Data_format = 1D COMPLEX
Dim_size = 26214
Dim_title = 13C
Dim_units = [ppm]
Dimensions = X
Site = ECX 400
Spectrometer = DELTA2_NMR
Field_strength = 9.389766[T] (400[MHz]
X_acq_duration = 1.04333312[s]
X_domain = 13C
X_freq = 100.52530333[MHz]
X_offset = 100[ppm]
X_points = 32768
X_prescans = 4
X_resolution = 0.95846665[Hz]
X_sweep = 31.40703518[kHz]
Irr_domain = 1H
Irr_freq = 399.78219838[MHz]
Irr_offset = 5[ppm]
Clipped = FALSE
Incomplete_copy = TRUE
Mod_return = 1
Scans = 321
Total_scans = 321
X_90_width = 7[us]
X_acq_time = 1.04333312[s]
X_angle = 30[deg]
X_atn = 6[dB]
X_pulse = 2.33333333[us]
Irr_atn_dec = 21.1[dB]
Irr_atn_noe = 21.1[dB]
Irr_noise = WALTZ
Decoupling = TRUE
Initial_wait = 1[s]
Noe = TRUE
Noe_time = 2[s]
Recvr_gain = 50
Relaxation_delay = 2[s]
Repetition_time = 3.04333312[s]
Temp_get = 80.1[dC]

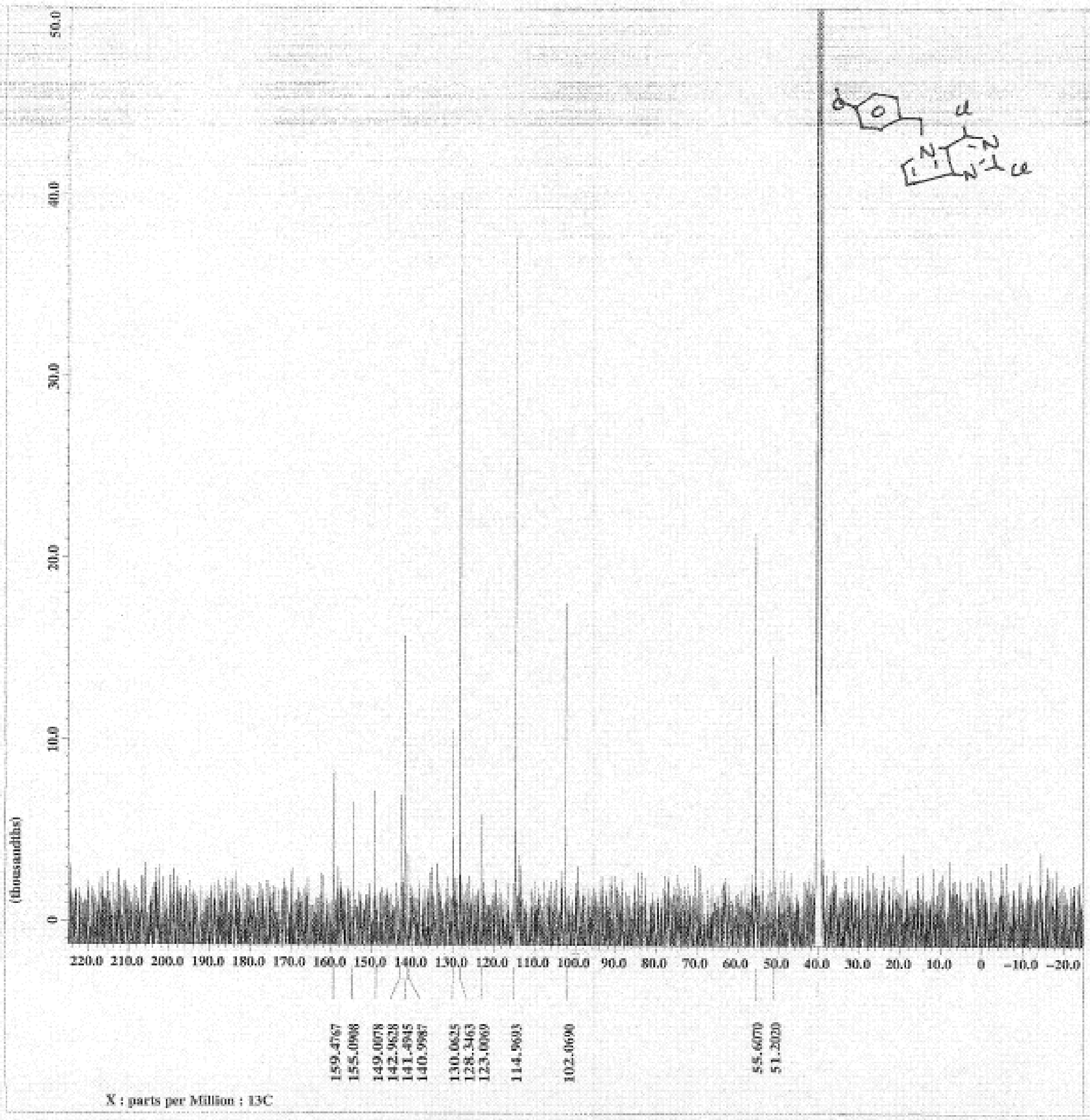

JEOL

Filename	= single_pulse_dec_copy
Author	= Seley
Experiment	= single_pulse_dec
Sample_id	= S#466542
Solvent	= DMSO-D6
Creation_time	= 11-OCT-2018 13:15:28
Revision_time	= 11-OCT-2018 13:16:26
Current_time	= 11-OCT-2018 13:16:29
Comment	= single pulse decouple
Data_format	= 1D COMPLEX
Dim_size	= 26214
Dim_title	= 13C
Dim_units	= [ppm]
Dimensions	= X
Site	= ECX 400
Spectrometer	= DELTA2_NMR
Field_strength	= 9.389766[T] (400[MHz]
X_acq_duration	= 1.04333312[s]
X_domain	= 13C
X_freq	= 100.52530333[MHz]
X_offset	= 100[ppm]
X_points	= 32768
X_prescans	= 4
X_resolution	= 0.95846665[Hz]
X_sweep	= 31.40703518[kHz]
Irr_domain	= 1H
Irr_freq	= 399.78219838[MHz]
Irr_offset	= 5[ppm]
Clipped	= FALSE
Incomplete_copy	= TRUE
Mod_return	= 1
Scans	= 354
Total_scans	= 354
X_90_width	= 7[us]
X_acq_time	= 1.04333312[s]
X_angle	= 30[deg]
X_atn	= 6[dB]
X_pulse	= 2.33333333[us]
Irr_atn_dec	= 21.1[dB]
Irr_atn_noe	= 21.1[dB]
Irr_noise	= WALTZ
Decoupling	= TRUE
Initial_wait	= 1[s]
Noe	= TRUE
Noe_time	= 2[s]
Recvr_gain	= 50
Relaxation_delay	= 2[s]
Repetition_time	= 3.04333312[s]
Temp_get	= 79.7[dC]

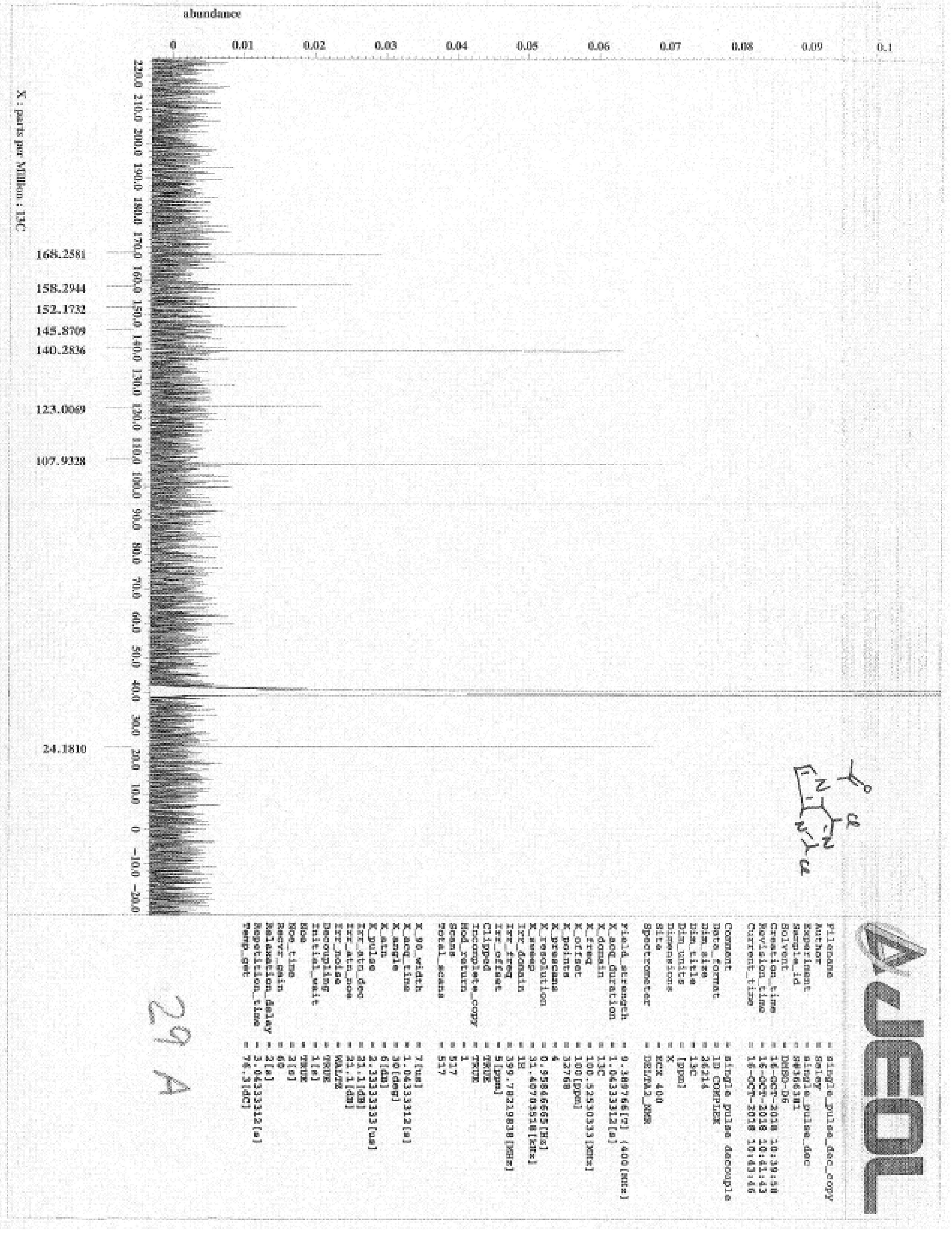
abundance
X : parts per Million : 13C
168.2581
158.2944
152.1732
145.8709
140.2836
123.0069
107.9328
24.1810
JEOL
29 A

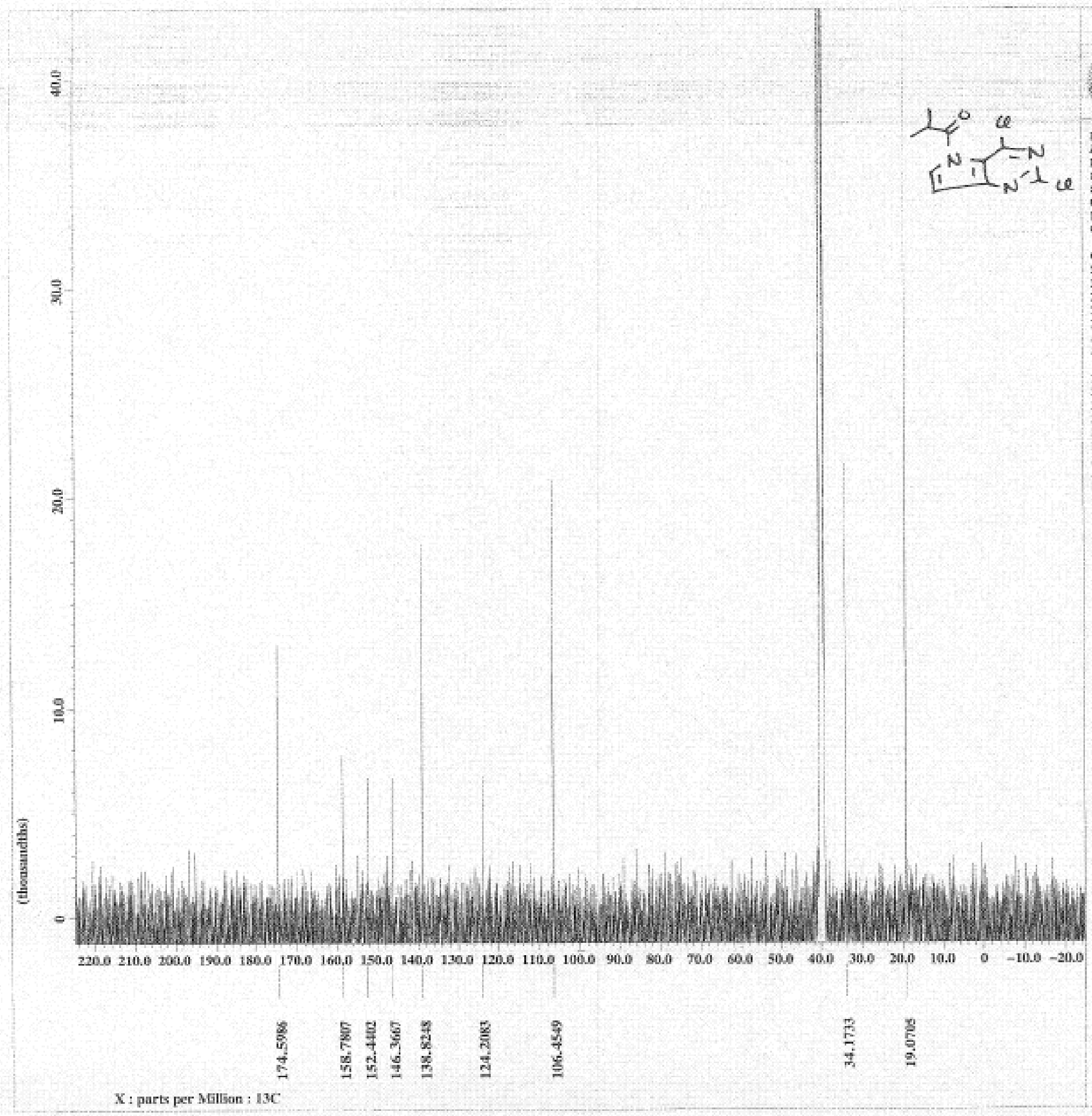

JEOL

Filename	=	single_pulse_dec_copy
Author	=	Seley
Experiment	=	single_pulse_dec
Sample_id	=	S#389423
Solvent	=	DMSO-D6
Creation_time	=	16-OCT-2018 11:10:18
Revision_time	=	16-OCT-2018 11:11:13
Current_time	=	16-OCT-2018 11:11:14
Comment	=	single pulse decouple
Data_format	=	1D COMPLEX
Dim_size	=	26214
Dim_title	=	13C
Dim_units	=	[ppm]
Dimensions	=	X
Site	=	ECX 400
Spectrometer	=	DELTA2_NMR
Field_strength	=	9.389766[T] (400[MHz]
X_acq_duration	=	1.04333312[s]
X_domain	=	13C
X_freq	=	100.52530333[MHz]
X_offset	=	100[ppm]
X_points	=	32768
X_prescans	=	4
X_resolution	=	0.95846665[Hz]
X_sweep	=	31.40703518[kHz]
Irr_domain	=	1H
Irr_freq	=	399.78219838[MHz]
Irr_offset	=	5[ppm]
Clipped	=	FALSE
Incomplete_copy	=	TRUE
Mod_return	=	1
Scans	=	415
Total_scans	=	415
X_90_width	=	7[us]
X_acq_time	=	1.04333312[s]
X_angle	=	30[deg]
X_atn	=	6[dB]
X_pulse	=	2.33333333[us]
Irr_atn_dec	=	21.1[dB]
Irr_atn_noe	=	21.1[dB]
Irr_noise	=	WALTZ
Decoupling	=	TRUE
Initial_wait	=	1[s]
Noe	=	TRUE
Noe_time	=	2[s]
Recvr_gain	=	50
Relaxation_delay	=	2[s]
Repetition_time	=	3.04333312[s]
Temp_get	=	77.5[dC]

29B

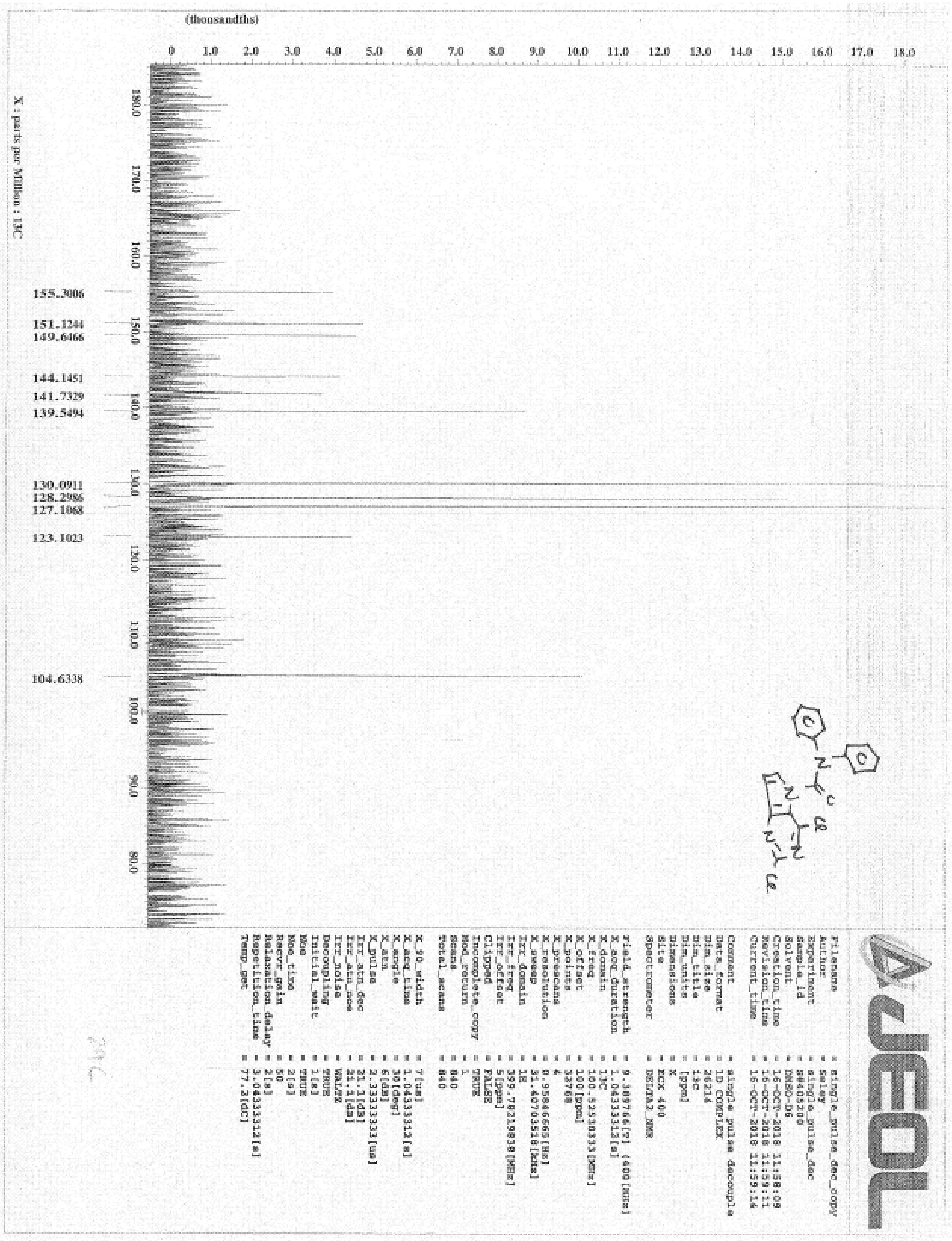
(thousandths)
X : parts per Million : 13C
155.3006
151.1244
149.6466
144.1451
141.7329
139.5494
130.0911
128.2986
127.1068
123.1023
104.6338
JEOL
Filename = single_pulse_dec_copy
Author = Seley
Experiment = single_pulse_dec
Sample_id = S#405200
Solvent = DMSO-D6
Creation_time = 16-OCT-2018 11:58:09
Revision_time = 16-OCT-2018 11:59:11
Current_time = 16-OCT-2018 11:59:14
Comment = single pulse decouple
Data_format = 1D COMPLEX
Dim_size = 26214
Dim_title = 13C
Dim_units = [ppm]
Dimensions = X
Site = ECX 400
Spectrometer = DELTA2_NMR
Field_strength = 9.389766[T] (400[MHz]
X_acq_duration = 1.04333312[s]
X_domain = 13C
X_freq = 100.52530333[MHz]
X_offset = 100[ppm]
X_points = 32768
X_prescans = 4
X_resolution = 0.95846665[Hz]
X_sweep = 31.40703518[kHz]
Irr_domain = 1H
Irr_freq = 399.78219838[MHz]
Irr_offset = 5[ppm]
Clipped = FALSE
Incomplete_copy = TRUE
Mod_return = 1
Scans = 840
Total_scans = 840
X_90_width = 7[us]
X_acq_time = 1.04333312[s]
X_angle = 30[deg]
X_atn = 6[dB]
X_pulse = 2.33333333[us]
Irr_atn_dec = 21.1[dB]
Irr_atn_noe = 21.1[dB]
Irr_noise = WALTZ
Decoupling = TRUE
Initial_wait = 1[s]
Noe = TRUE
Noe_time = 2[s]
Recvr_gain = 50
Relaxation_delay = 2[s]
Repetition_time = 3.04333312[s]
Temp_get = 77.2[dC]

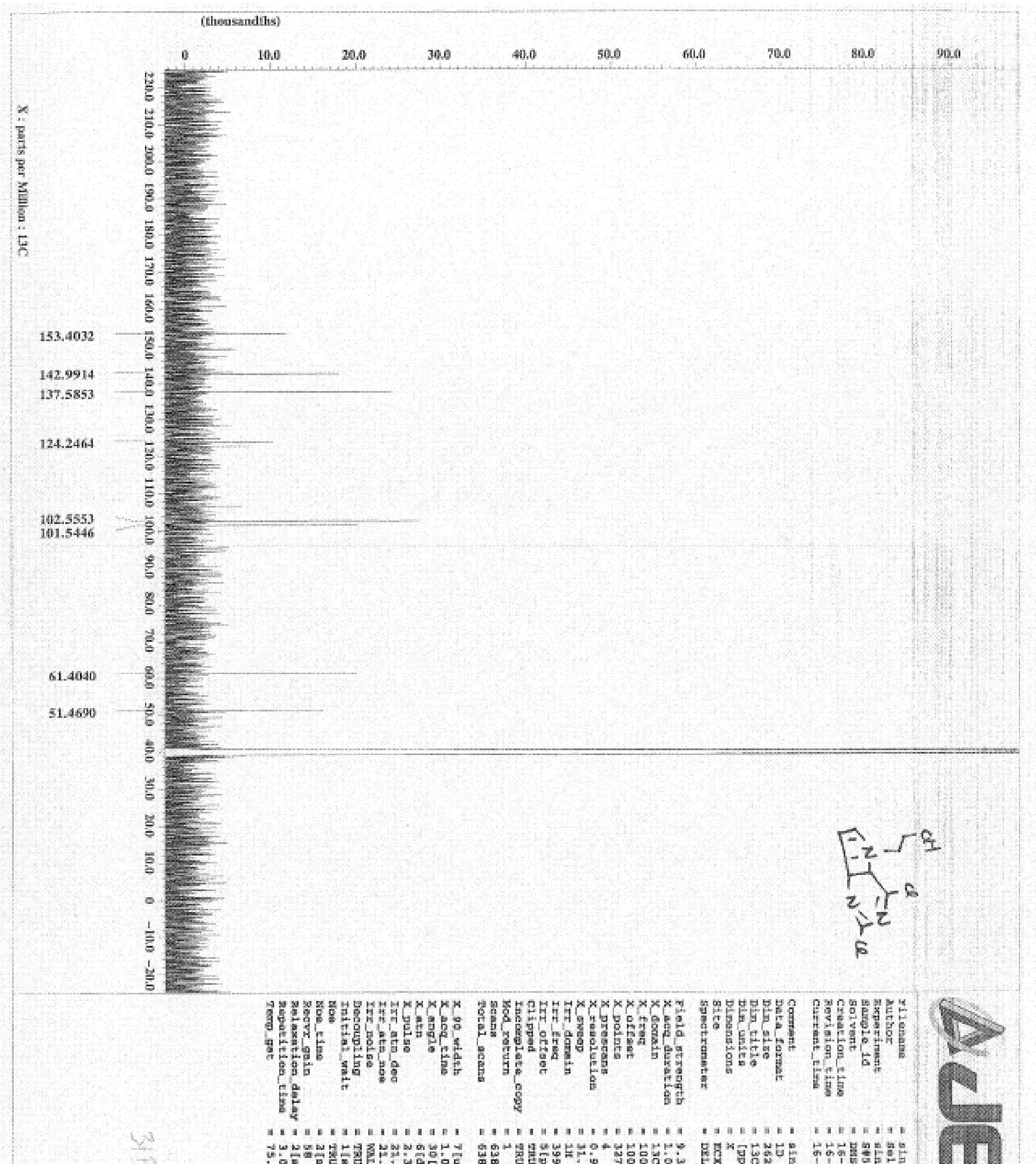

(thousandths)
0
10.0
20.0
30.0
40.0
50.0
60.0
70.0
80.0
90.0
220.0 210.0 200.0 190.0 180.0 170.0 160.0 150.0 140.0 130.0 120.0 110.0 100.0 90.0 80.0 70.0 60.0 50.0 40.0 30.0 20.0 10.0 0 −10.0 −20.0
153.4032
142.9914
137.5853
124.2464
102.5553
101.5446
61.4040
51.4690
X : parts per Million : 13C
JEOL
Filename = single_pulse_dec_copy
Author = Seley
Experiment = single_pulse_dec
Sample_id = S#583555
Solvent = DMSO-D6
Creation_time = 16-OCT-2018 16:47:25
Revision_time = 16-OCT-2018 16:49:17
Current_time = 16-OCT-2018 16:49:18
Comment = single pulse decouple
Data_format = 1D COMPLEX
Dim_size = 26214
Dim_title = 13C
Dim_units = [ppm]
Dimensions = X
Site = ECX 400
Spectrometer = DELTA2_NMR
Field_strength = 9.389766[T] (400[MHz]
X_acq_duration = 1.04333312[s]
X_domain = 13C
X_freq = 100.52530333[MHz]
X_offset = 100[ppm]
X_points = 32768
X_prescans = 4
X_resolution = 0.95846665[Hz]
X_sweep = 31.40703518[kHz]
Irr_domain = 1H
Irr_freq = 399.78219838[MHz]
Irr_offset = 5[ppm]
Clipped = TRUE
Incomplete_copy = TRUE
Mod_return = 1
Scans = 638
Total_scans = 638
X_90_width = 7[us]
X_acq_time = 1.04333312[s]
X_angle = 30[deg]
X_atn = 6[dB]
X_pulse = 2.33333333[us]
Irr_atn_dec = 21.1[dB]
Irr_atn_noe = 21.1[dB]
Irr_noise = WALTZ
Decoupling = TRUE
Initial_wait = 1[s]
Noe = TRUE
Noe_time = 2[s]
Recvr_gain = 58
Relaxation_delay = 2[s]
Repetition_time = 3.04333312[s]
Temp_get = 75.9[dC]
